# Interpreting vibrational circular dichroism spectra: the Cai•factor for absolute configuration with confidence

**DOI:** 10.1186/s13321-023-00706-y

**Published:** 2023-03-21

**Authors:** Jonathan Lam, Richard J. Lewis, Jonathan M. Goodman

**Affiliations:** 1 Centre for Molecular Informatics, Yusuf Hamied Department of Chemistry, Lensfield Road, Cambridge, CB2 1EW UK; 2 Department of Medicinal Chemistry, Research & Early Development, Respiratory & Immunology, BioPharmaceuticals R&D, AstraZeneca, 43183 Mölndal, Sweden

**Keywords:** VCD data, Absolute configuration, VCD, Structure determination

## Abstract

Vibrational circular dichroism (VCD) spectroscopy can generate the data required for the assignment of absolute configuration, but the spectra are hard to interpret. We have recorded VCD data for thirty pairs of small organic compounds and we use this database to validate a method for the automated analysis of VCD spectra and the assignment of absolute configuration: the *Cai•factor* (Configuration: absolute information). The analysis of the data demonstrates that the procedure is a reliable and time-efficient method for determination of absolute configuration, which gives both the assignment and a measure of confidence in the outcome, even when the spectra are imperfect. The majority of molecules tested have a high confidence score and all of these have the correct assignment.

**Supplementary Information:**

The online version contains supplementary material available at 10.1186/s13321-023-00706-y.

## Introduction

Absolute configuration is of central importance to the function and efficacy of drugs and biomolecules. Enantiomers of drug molecules usually show different efficacies at their targets. Knowledge of the structure of the active enantiomer can help in the design of improved therapeutic molecules. Similarly, the synthetic chemist may need to determine the absolute stereochemistry of an early intermediate in a synthetic sequence with the aim of making the synthesis of a complex chiral molecule as efficient as possible. X-ray crystallography [1] is an established method for determination of absolute configuration, but requires crystalline material of sufficient quality to give a definitive result.

As an alternative, spectroscopic chiroptical methods, such as vibrational circular dichroism (VCD) are of growing importance and can obtain absolute stereochemical information on molecules in solution [2]. VCD has been used in the determination of absolute configuration of complex molecules for more than two decades [3, 4]. Recent developments have been reviewed [5, 6], and the approach has been applied to many structures [7–9]. As a recent example [10], a combination of total synthesis, VCD and ECD was used to reassign the correct stereochemistry for the complex alkaloid Pilemartine A which is not crystalline and therefore difficult to establish.

The interpretation of VCD spectra is not a straightforward task, particularly if the spectra are noisy. Unlike IR and NMR spectra, there are no rules for directly interpreting VCD spectra. Therefore, a comparison between experiment and calculation must be made in order to gain comprehensible information from the data. Standard DFT computational chemistry programs, such as Jaguar [11] and GAUSSIAN [12], are capable of calculating VCD signals from representative molecular conformations. Even with these tools the interpretation of VCD spectra is challenging.

**Fig. 1:**
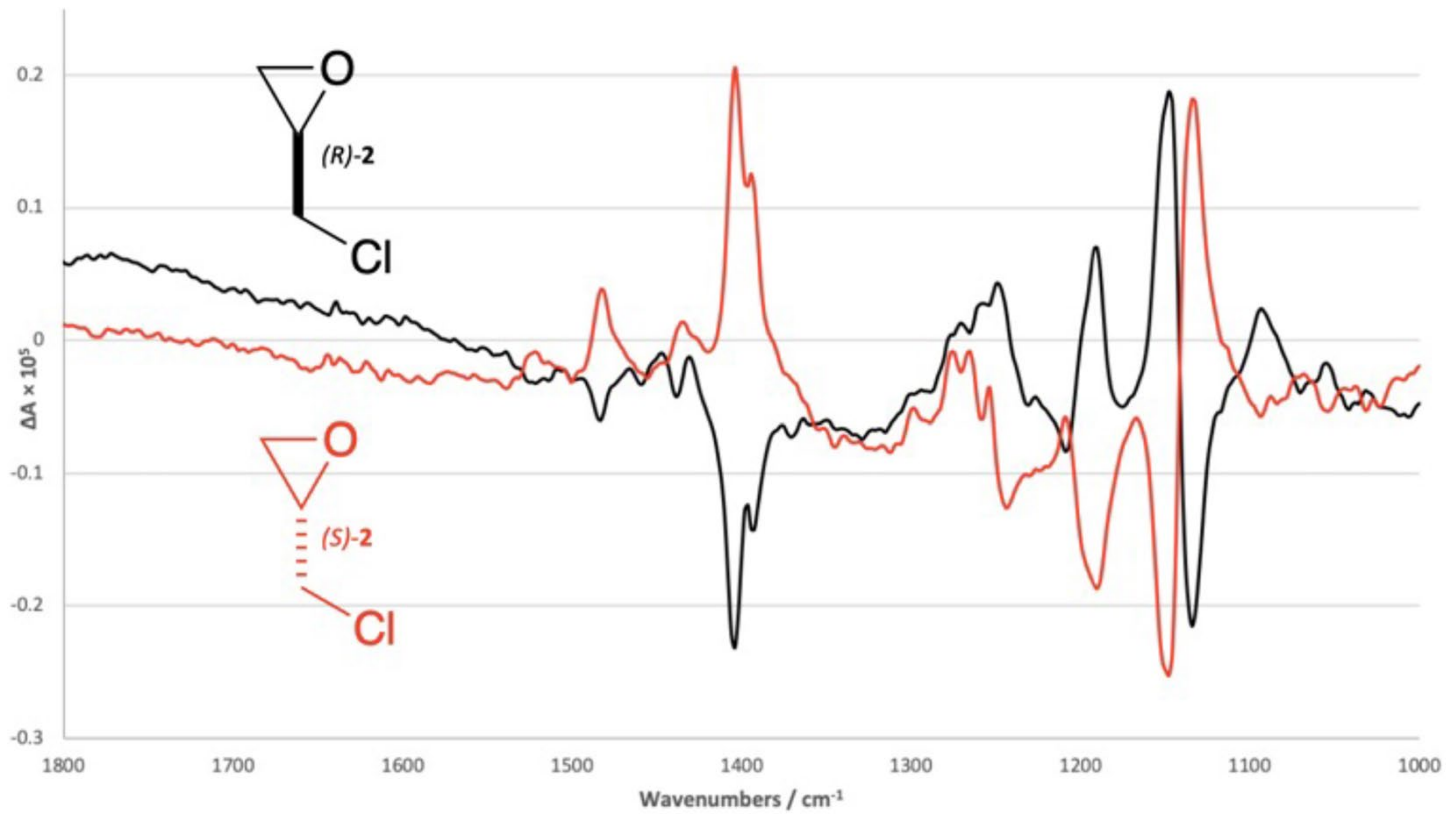
VCD spectra for (R)-2-(chloromethyl)oxirane (black line, *(R)-*2) and (S)-2-(chloromethyl)oxirane (red line, *(S)-*2). The two spectra would be mirror images if the experimental error in the measurements could be completely eliminated

Figure 1 shows two VCD spectra, corresponding to the enantiomers of 2-(chloromethyl)oxirane. Perfect spectra of enantiomers, with no noise and flat baselines, would be reflected in the line ∆A = 0 but otherwise be identical. Perfect VCD spectra of racemic or achiral substances show no signals. These experimental spectra are approximate mirror images. The signals can appear both above and below the baseline, the baseline will not always be completely flat, and intense peaks may saturate the signal. Extending the time used for data collection is an effective way of improving the quality of spectra, as is acquiring data for a blank spectrum of the solvent, or making measurements on both enantiomers of the substrate. In practice, however, the interpretation of imperfect spectra may well be needed, as pressure on resources may make extended data acquisition times challenging, contemporaneous blank spectra may not have been recorded, or else the enantiomer of the substrate was not available.

Shen, et al. developed SimIR/VCD to interpret these data [13]. The process was tested on α-pinene and carvone as well as several other molecules for which the structure was not revealed, and found to be an effective guide. Another algorithm, CompareVOA, was developed by Bultinck, et al*.* [14] and was tested on a database of 83 molecules, although data is reported only for 3R-methylcyclohexanone and R-limonene. The paper reports that the use of the large database made it possible to get a better feeling for the quality of the assignment of absolute configuration. More recent papers focus on the use of VCD to look at conformational flexibility. Sherer, et al*.* [15] used the substantial computational resources of the Merck Research Laboratories to analyse complete flexible molecules rather than considering them one fragment at a time and concluded that combining a qualitative visual analysis with the quantitative results of CompareVOA is effective. Another study [16], focussed on citronellal and dehydroquinidine, used the SimIR/VCD program to demonstrate that it is possible to analyse the VCD spectra of very flexible molecules. Inspired by these studies, and building on our analyses of NMR spectra, [17] we have developed an open-source, program, Cai•factor (Configuration: absolute information), which makes possible the automated assignment of absolute configuration from VCD spectra with an estimate of the confidence that may be had in the results.

## Results

### Experimental results

**Fig. 2:**
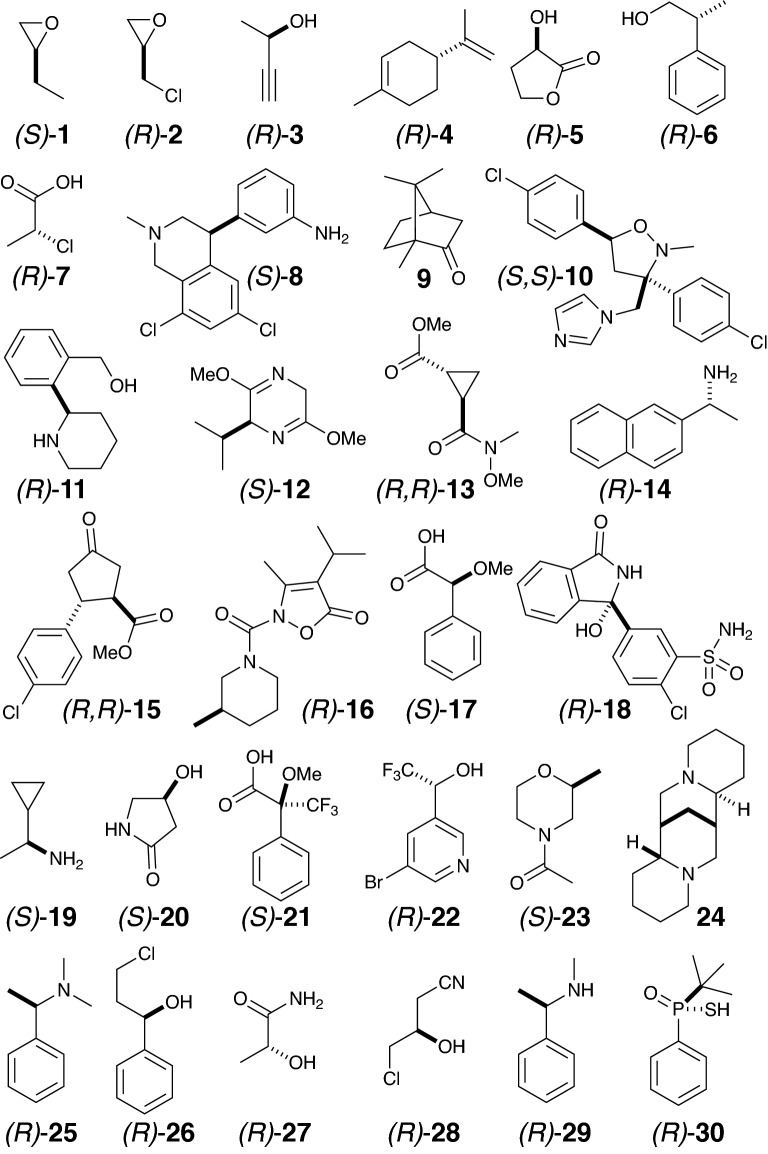
The compounds studied. All VCD data available in the ESI

The thirty enantiomeric pairs of compounds (Fig. 2) were selected to have varying degrees of conformational flexibility, ranging from structures with only one stable conformation to those with many low-energy conformers. Several drug compounds and drug precursor molecules were used, including some with less-common functionalities, such as the sulfonamide group in chlorthalidone, 18. The compounds were dissolved in chloroform-d1 or DMSO-d6. Sample concentrations were chosen such that absorption bands were clearly visible in the IR and VCD spectra whilst not being so concentrated that intermolecular effects gain importance. IR and VCD spectra were recorded on a BioTools ChiralIR-2X Spectrometer (1–23) and a Bruker TENSOR FTIR spectrometer with a PMA50 module for polarization modulated measurements (1–7, 24–30). Solutions of the samples were held in a BaF_2_ transmission cell with a path length of 100 µm. Both IR and VCD spectra were recorded at a spectral resolution of 4 cm^−1^ by accumulating about 20 000 scans over six hours.

### Calculation of VCD spectra

In order to calculate the spectra, each molecule was constructed in the Maestro molecular modelling program and a Macromodel [18] conformational search was run. All conformational searches were performed using the “Mixed torsional/Low-mode sampling” method, with the torsional sampling option set to “Extended”, and the Merck Molecular Force Field (MMFF) to determine the energy of each conformer. The OPLS3e force field was found to give similar results. In order for the conformational search to model that of the VCD experiment as closely as possible, the chloroform solvent model was applied. A suitable number of steps was chosen for each conformational search such that conformations 10 kJ mol^−1^ or less above the ground state were all found five times or more.

This process was applied to the thirty molecules in Fig. 2. The first seven spectra were recorded on both Bruker and BioTools instruments, so 37 pairs of spectra were available. The calculations were run using four levels of theory: B3LYP/6-31G(d,p), B3LYP/cc-pVTZ, B3PW91/6-31G(d,p), and B3PW91/cc-pVTZ, as implemented in the Jaguar [11] software package on all of the low energy conformations generated by a molecular mechanics conformation search, both as single point calculations on the molecular mechanics geometries and after re-minimisation at the DFT level. Details are given in the ESI.

### Development of the Cai•factor

It was not clear how best to analyse the large amount of data that was now available. To investigate the many possibilities, we wrote a program, Cai, which enabled us to process this in many different ways. Cai takes the experimental and computational data as its input, and follows the instructions in a command file to compare the calculation and the experiment. The command file makes it easy to adjust the details of the calculation, including the range of frequencies that should be considered, the temperature used to calculate the Boltzmann average of multiple conformations, and other parameters. The program, which is written in Python, is available on Github: github.com/Jonathan-Goodman/cai-factor. The program’s output is a record of the calculation, the Cai•factor for the confidence in the assignment, and a graphical record of the experimental and calculated data.

The accuracy of DFT vibrational frequency calculations is limited by the neglect of the anharmonicity of molecular vibrations. The results contain systematic errors, and scale factors are required to improve the match between calculated and observed VCD spectra [19]. Small conformational changes may also cause a sign change in some VCD signals. Such signals are known as non-robust vibrational modes [20]. Solute–solute interactions, such as intermolecular hydrogen bonding and dimerization, and solvent–solute interactions, can introduce changes to the experimental VCD spectrum which are missed in calculations. That the technique is sensitive to these subtle phenomena is part of the power of VCD. A procedure is needed which can robustly predict absolute configuration with a high degree of confidence for a wide range of molecules. It is conceivable that all these sources of uncertainty could be identified and accurately included in calculations; however, a generalised method for predicting VCD that is too computationally demanding results in wasteful use of resources to give only a slight increase in confidence in configuration assignments. In order for VCD-based absolute configuration determination to be applicable over a broad range of compounds without unnecessary use of computational resources, a technique capable of assigning absolute configuration, despite the uncertainties caused by these complexities, is needed. In this work, the goal is the assignment of the correct enantiomer rather than the generation of a perfect fit for the experimental data and the requirement for an unblemished spectrum. It may be possible to give a confident assignment of absolute configuration even from imperfect experimental data and simulations containing significant approximations. Spectra can be calculated with many different functionals and basis sets. The B3LYP/6-31G* [21] combination is widely used and fast, although less accurate than larger basis sets, such as cc-pVTZ [22], which offer greater accuracy at the expense of longer computational times. Our earlier studies of NMR spectra [23, 24] have demonstrated that the highest computational levels do not always give the best discrimination between isomers. It is not necessarily the case, therefore, that functionals and procedures with greater computational costs offer good value in terms of increased accuracy of enantiomer assignment from the position and intensity of the calculated VCD transitions.

The calculated VCD spectra are generated as a series of sharp lines for each conformation, whereas experimental spectra show broad peaks. The calculated spectra were therefore transformed to more closely resemble the experimental ones by combining the peaks for the different conformations using Boltzmann weighting from the calculated energies, scaling the calculated wavenumbers, and broadening the lines. Initially, we used a scaling factor of 0.975, and a Lorentzian broadening with a half width at half maximum (HWHM) of 5 cm^−1^. As we describe below, the experimental dataset was used to test and improve these initial values. These values can all be set as parameters in the command file.

The difference between the simulated and the experimental spectra were assessed using a multiplicative scoring method. Because of the possibility of sign changes arising from small changes in conformation, signals with the highest VCD intensity are not necessarily the best indicators of a compound’s absolute configuration. For each point along the experimental wavenumber axis, the calculated (c) and observed (o) values are multiplied, giving a positive outcome when they have the same sign and a negative outcome otherwise. These products are then summed (ΣCO) to give the overall outcome, either positive or negative. This sum is then scaled by the geometric mean of the sums of the squares of the calculated value at each point (ΣCC) and the observed value at each point (ΣOO).

The overall result, which is a modification of the SimVCD integral [13], is given by the expression:

$$\mathbf{s}\mathbf{i}\mathbf{m}\mathbf{i}\mathbf{l}\mathbf{a}\mathbf{r}\mathbf{i}\mathbf{t}\mathbf{y}\,\mathbf{f}\mathbf{a}\mathbf{c}\mathbf{t}\mathbf{o}\mathbf{r}=\frac{\boldsymbol{\Sigma }{\varvec{c}}{\varvec{o}}}{\sqrt{\boldsymbol{\Sigma }{\varvec{c}}{\varvec{c}}\boldsymbol{\Sigma }{\varvec{o}}{\varvec{o}}}}$$

**Fig. 3:**
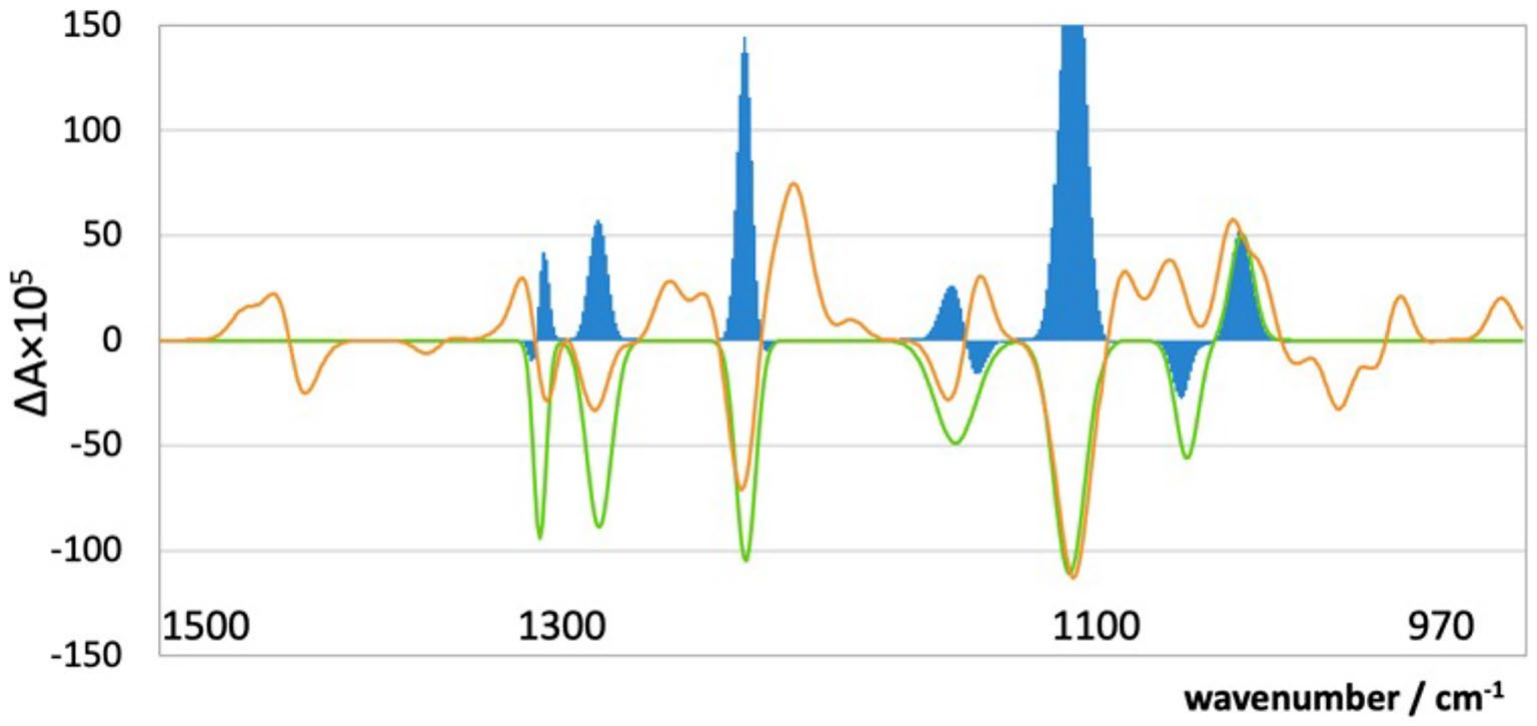
Demonstration of the multiplicative scoring method for the calculated (green trace) and observed (orange trace) VCD spectra of sparteine (24). Blue areas are representative of the multiplicative score

The method is illustrated in Fig. 3. In this example, the blue areas represent the calculated-observed products. The blue bars are predominantly on the positive side of the axis, and so, in this example, the experimental spectrum shows a good match with the absolute configuration used in the calculation. A large, positive, similarity factor suggests that the calculated and the experimental molecules have the same absolute configuration, whereas a negative outcome suggests they have opposite configurations.

In addition to this graphical output, the Cai program also produces a log file with a more detailed numerical analysis. In this example, two different calculated conformations fell within the default 10 kJ mol^−1^ energy cut-off and their spectra were combined using Boltzmann weighting and the default temperature of 300 K. The energy cut-off and the temperature are parameters which can be set in the command file but the default settings are usually appropriate. The calculation was run for the experimental spectra of the two enantiomers separately, as well as for the combined spectrum which is created by subtracting one spectrum from the other, a process which helps correct any non-linearity in the baseline for the measurements. In general, a combined spectrum, if available, gives higher confidence than a single enantiomer spectrum. If experimental data for just one enantiomer is available, a blank spectrum, can also be used to correct for the baseline variation.

Wavenumber scaling is applied to the DFT-calculated spectra in order to ensure best overlap with the experimental data. Initially, we scanned through a range of scale factors from 0.950 to 1.000, in increments of 0.005, to optimize the absolute value of the similarity factor. Having determined that the best results come towards the middle of this range, it was not necessary to explore more extreme values, and a value of 0.975 was found to be a good choice for B3LYP/6-31G(d,p); 0.980 was preferred for B3PW91/cc-pVTZ (details in SI). The Cai program calculates the similarity factor with the chosen default scaling factor (specified in the input file), and also investigates whether the match can be improved by adjusting the value.. For the example in Fig. 3, a scaling factor of 0.975 gives a similarity factor of 68, and a scaling factor of 0.976 gives a slightly improved value, which is still 68 to two significant figures. In a few examples, a large change to the scaling factor significantly improves the match. In such cases, the program gives a warning and it is useful to check the comparison of the spectra by eye. It is sometimes obvious that one large peak is dominating the spectrum which is controlling the scaling factor and the outcome. In such cases, this peak may be interpreted correctly as the dominant feature of the spectrum, or it may be appropriate to omit it from the spectrum by reducing the range of wavenumbers that the program considers.

The two input spectra are also analysed individually, and the results are reported in the log file. In this example, spectrum A has a similarity factor of 34 and spectrum B has a similarity factor of 38; both of these results give reasonable confidence in the assignment and are consistent with the combined analysis.

### Analysis of experimental and computational results

**Fig. 4:**
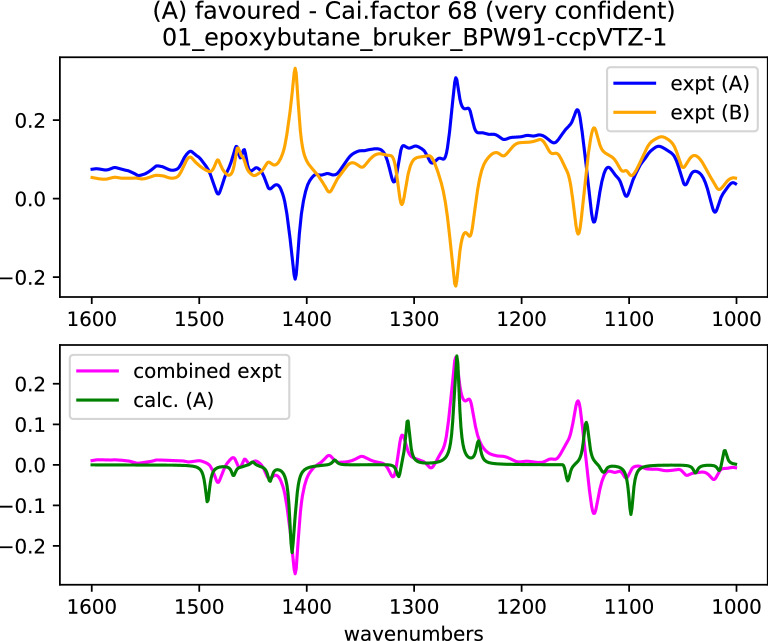
The output of Cai for molecule (S)-1. In this example, separate spectra were measured for each enantiomer (orange and blue lines in the top panel). The combined spectrum (purple, lower panel) was compared with the calculation (green). The vertical axis is the absorbance in arbitrary units. The fit in this example is reasonably good and there is a high Cai•factor

Initially, we did not know what the optimal choice of parameters would be. Therefore, we ran the program for all our experimental and calculated data and gathered the outputs into a single spreadsheet (available in the ESI) which enabled us to compare the possibilities. The wavenumber range 1000 cm^−1^ to 1600 cm^−1^ was appropriate in most cases, even though a wider range was available experimentally. The region below 1000 cm^−1^ did not add useful information to the analysis in most cases. In many molecules, there is a double-bond band above 1600 cm^−1^ which absorbs so strongly that the signal saturates. In the case of carbonyls the frequency is not always well calculated. This can cause a problem if the VCD signal is both intense and biphasic. This is most easily addressed by omitting the signals by restricting the range of frequencies considered, although the Cai program command file can be set to include it, if required. If experimental spectra for both enantiomers of the substrate are available, the program combines the two experimental measurements to calculate the assignment. If only one experimental spectrum is available, which is a common situation, the program can still calculate the assignment. If a spectrum of the pure solvent (“blank spectrum”) is available (solvent or racemate for example) this can also be included, as it records the baseline of the spectrometer and the cell. This is most effective when done at the same time as acquiring the experimental data of the sample. An example of the output is shown in Fig. 4. In this case, two reasonably good spectra are available, and the absolute configuration can be assigned with high confidence. Visual inspection of the spectra would probably come to the same conclusion as the program, although there is a region of the spectrum in the 1100 cm^−1^ to 1150 cm^−1^ region where the calculation and the experiment appear to disagree.

**Fig. 5:**
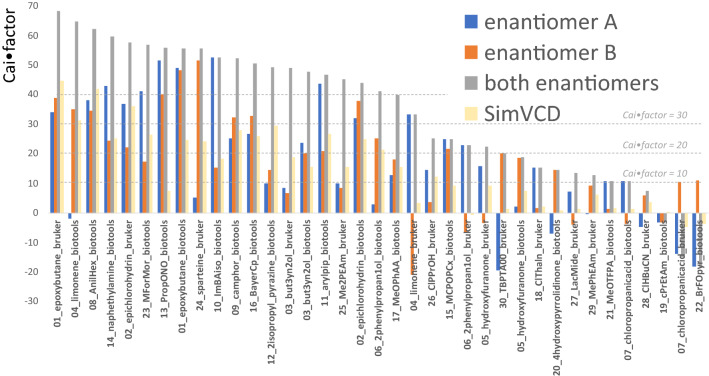
The results of the studies of our dataset of thirty compounds, ordered by Cai•factor for the calculation based on the spectra of both enantiomers (grey bars). The blue and the orange bars are for the enantiomers taken separately. Enantiomer A is the molecule illustrated in Fig. 2 and enantiomer B is its mirror image. The figure also shows the results of a SimVCD calculation, on the combined experimental data, calculated using the website: http://simvcd.net/

The program calculates similarity factors for spectra separately and combined, with fixed and optimised scaling factors, and distils the results into a single Cai•factor, which is the most confident of the assignments after concerns about unusual scaling factors and inconsistent data have been taken into account. Analysis of the Cai•factors for all thirty of our test molecules (details in the ESI) showed that the highest average score is reached by geometry optimization at the B3PW91/cc-pVTZ level of theory. Despite the resource-intensive calculation, we verified that the triple-zeta basis set gives the most accurate outcome out of the various methods sampled. For each method, the match scores were calculated both using single point DFT calculations on the molecular mechanics geometries, and by optimising these geometries using DFT, which is a much more computationally demanding process. The Cai•factors are compared in Fig. 5. All results above 20 are correct, in our testing but a few incorrect assignments had values less than but close to 20. As a result, we chose 20 to be the value at which there can be some confidence in the result. In practice, we treat results from 20–30 with caution. Geometry optimization using DFT gives a significant increase in the Cai•factor, but greatly increases the cost of the analysis.

The outcomes of SimVCD calculations are also given in Fig. 5. A value of more than 0.2 is required for a confident assignment, according to the SimVCD website http://simvcd.net. Thirteen of the spectra lead to confidence on the basis of the SimVCD measure.

Whenever possible, VCD spectra for both enantiomers of each compound were used. For cases where only a single enantiomer was available, the same method can be applied. By taking the VCD spectra of enantiomeric pairs of compounds and analysing each enantiomer separately, the case of assigning the absolute configuration of a compound where only a single enantiomer was available could be simulated.

The results are also included in Fig. 5, as the orange and the blue bars. What quality is good enough? The Cai•factor gives an automated interpretation of this. On the basis of the data in Fig. 5, a Cai•factor of less than ten means that it is hard to draw a conclusion. This may well mean that the spectrum is of low quality, either because it is noisy and the signal is weak or else because there are few distinct peaks in the spectra. Cai•Factors between ten and twenty are usually correct, but there are two examples of ten in this range for which they are misleading for the analysis based on both enantiomers, and three out of sixteen where they are misleading for a single enantiomer measurement. Above a Cai•factor of twenty, the assignments are all correct for both enantiomer and single enantiomer calculations, with the single exception of 04_limonene_bruker which has a Cai•factor of 21 for the incorrect assignment. The low quality of the measurement leads to the outcome. At Cai•factors above thirty, the visual relationship between the calculation and the experiment may not be very close (see ESI) but a fairly high level of confidence may still be had in the assignments. Above forty, the visual assignment is usually very clear, justifying a high level of confidence. The Cai•factor, therefore, increases the confidence that may be had in a visual assessment of the correspondence between the calculation and the experiment, and may be used automatically without the need for a scientist to make this human-resource-intensive assessment.

### Analysis of three chiral drug molecules

**Fig. 6:**
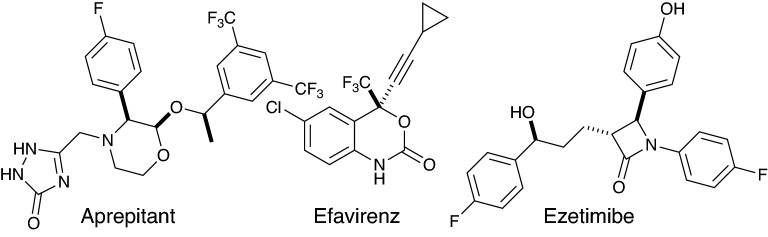
Efavirence, Ezetimibe and Aprepitant

We tested the process by analysis of three marketed chiral drug molecules, Aprepitant, Efavirenz and Ezetimibe (Fig. 6) These molecules are three of the more challenging examples used by Sherer [15] to test their systematic approach to conformational sampling in calculation of VCD spectra. Full details are given in the ESI.

**Aprepitant**: A Cai•factor of 35 (fairly confident) was calculated in favour of the known stereochemistry. An optimized scale factor of 0.984 gives a Cai•factor of 40.

**Efavirenz**: A Cai•factor of 4 (uncertain) was calculated but this rose to 47 for the known stereochemistry at a scale factor of 0.997. In order to decide whether to accept the unusual scale factor it is instructive to view the fit produced by the Cai algorithm (Additional file 1: Fig. S2, ESI). This shows that whereas the majority of small peaks in the experimental spectrum are fit well at a scale factor of 0.98, two intense peaks at 1250 and 1265 cm^−1^ are not, even though the qualitative pattern fits well. These intense peaks dominate the similarity factor integral and account for the low score. The decision can be made to accept the higher scale factor of 0.997. The prominent peak between 1700 and 1800 cm^−1^ is not present in the calculation and so has no net effect on the Cai•factor. The alignment of the feature between 1200 and 1300 cm^−1^ is the determining element in the analysis.

**Ezetimibe**: A Cai•factor of 21 (cautiously confident) was calculated for the known stereochemistry. An optimized scale factor of 0.977 gives a Cai•factor of 23. We noted that in the minimum energy conformation (see ESI), the sidechain was extended, whereas the minimum energy conformation reported by Sherer [15] showed a hydrogen bond from the side-chain hydroxyl to the amide carbonyl. We were able to reproduce these results by repeating the calculations at the B3LYP/6-31G* level in the gas phase using the same set of 130 starting conformations. The minimum energy conformation was then very similar to that described by Sherer and the Cai•factor increased to 32 (fairly confident) and to 37 with an optimised scale factor of 0.966.

### Observations on further analyses

We have also applied the Cai•factor analysis retrospectively to 22 recent AstraZeneca molecules where we determined absolute stereochemistry using a visual method described previously [25]. Conformational searches and calculations followed the method described above, with calculations performed at either B3LYP/6-31 g* or B3PW91/cc-pVTZ levels. Molecules ranged in size between 250 and 500 MW with a range of flexibilities. Visual assessments resulted in an assessment of no match, possible match, reasonable match, good match or excellent match. The default scale factors were B3PW91/cc-pVTZ 0.98 and B3LYP/6-31 g* 0.975. Sixteen results had a Cai•factor greater than 30, two in the range 20–30 and three 10–20. For the results with a Cai•factor below 30, in four cases the visual inspection had also resulted in a cautious match with one case being designated a good match. For one of the 22 results, the Cai•factor made a possible assignment, which was consistent with other experimental evidence, when no match was made visually. For the other 21 results, all agreed with the visual assessment of stereochemistry.

## Conclusions

We present a new process to analyse VCD data: the Cai•factor. The procedure is straightforward to implement and has led to the automated interpretation of thirty sets of enantiomeric spectra. The process is able to give an interpretation and confidence level even for imperfect data, or when the assignment cannot be made visually. Use of the B3PW91 functional and cc-pVTZ basis set is recommended for the most reliable VCD assignments. The method has been applied to three compounds outside the original dataset returning confident assignment of absolute stereochemistry. It has also been successfully applied to more than twenty additional compounds within AstraZeneca. GitHub: github.com/Jonathan-Goodman/cai-factor.

## Supplementary Information

**Additional file 1: Figure S2.** Match of experimental and calculated VCD spectra for Efavirenz at a scale factors of (a) 0.975 and (b) 0.997.

## Data Availability

The program is available on GitHub: github.com/Jonathan-Goodman/cai-factor Details of the experiments and calculations are in the Supporting Information.
