# Supplementary material for: Interpreting vibrational circular dichroism spectra: the Cai•factor for absolute configuration with confidence
Source: J Cheminform. 2023 Mar 21;15:36. doi: 10.1186/s13321-023-00706-y (PMC10031863; doi:10.1186/s13321-023-00706-y)
Supplement: Supplementary file 1 — Additional file 1: Figure S2. Match of experimental and calculated VCD spectra for Efavirenz at a scale factors of (a) 0.975 and (b) 0.997. [file 13321_2023_706_MOESM1_ESM.docx]

Interpreting Vibrational Circular Dichroism Spectra: the Cai•Factor for Absolute Configuration with Confidence

Jonathan Lam,^1^ Richard J. Lewis^2^ and Jonathan M. Goodman^1^*

1. Centre for Molecular Informatics, Yusuf Hamied Department of Chemistry, Lensfield Road, Cambridge, CB2 1EW, UK

2. Medicinal Chemistry, Research & Early Development, Respiratory & Immunology, BioPharmaceuticals R&D, AstraZeneca, Gothenburg, Sweden

Additional information

[Experimental details 2](#_Toc92277946)

[Baseline correction 3](#_Toc92277947)

[Full reference for Gaussian 3](#_Toc92277948)

[Molecules and VCD data 3](#_Toc92277949)

[Molecule 01: epoxybutane 5](#_Toc92277950)

[Molecule 02: epichlorohydrin 6](#_Toc92277951)

[Molecule 03: but3yn2ol 7](#_Toc92277952)

[Molecule 04: limonene 8](#_Toc92277953)

[Molecule 05: hydroxyfuranone 9](#_Toc92277954)

[Molecule 06: 2phenylpropan1ol 10](#_Toc92277955)

[Molecule 07: chloropropanicacid 11](#_Toc92277956)

[Molecule 08: AnilHex 12](#_Toc92277957)

[Molecule 09: camphor 12](#_Toc92277958)

[Molecule 10: ImBAiso 13](#_Toc92277959)

[Molecule 11: arylpip 13](#_Toc92277960)

[Molecule 12: 2isopropyl: pyrazine 14](#_Toc92277961)

[Molecule 13: PropONO 14](#_Toc92277962)

[Molecule 14: naphethylamine 15](#_Toc92277963)

[Molecule 15: MCPOPCx 15](#_Toc92277964)

[Molecule 16: BayerCp 16](#_Toc92277965)

[Molecule 17: MeOPhAA 16](#_Toc92277966)

[Molecule 18: ClThaln 17](#_Toc92277967)

[Molecule 19: cPrEtAm 18](#_Toc92277968)

[Molecule 20: 4hydroxypyrrolidinone 18](#_Toc92277969)

[Molecule 21: MeOTFPA 19](#_Toc92277970)

[Molecule 22: BrFOpyr 19](#_Toc92277971)

[Molecule 23: MForMor 20](#_Toc92277972)

[Molecule 24: sparteine 20](#_Toc92277973)

[Molecule 25: Me2PEAm 21](#_Toc92277974)

[Molecule 26: ClPPrOH 21](#_Toc92277975)

[Molecule 27: LacMide 22](#_Toc92277976)

[Molecule 28: ClHBuCN 22](#_Toc92277977)

[Molecule 29: MePhEAm 23](#_Toc92277978)

[Molecule 30: TBPTA00 23](#_Toc92277979)

[Aprepitant 24](#_Toc92277980)

[Efavirenz 28](#_Toc92277981)

[Ezetimibe 34](#_Toc92277982)

# Experimental details

The chemicals were purchased from commercial sources, and experimental VCD spectra measured on a Biotools ChiralIR-2X instrument in CDCl_3_ (Efavirenz) or DMSO-d6 (Aprepitant and Ezetimibe), using the same concentrations as the literature precendent. A blank spectrum of solvent in the same cell was also collected. Macromodel (Schrodinger Inc.) was used to perform a conformational search using the OPLS3e force field and Mixed torsional / Low mode sampling and conformations within 21.0 kJ.mol^-1^ were retained. This resulted in 68 conformations for Aprepitant, 10 for Efavirenz and 130 for Ezetimibe. These conformations were then minimized and VCD spectra calculated in Gaussian16 at the B3PW91/cc-pVTZ level of theory using gas phase calculations for Efavirenz and a PCM DMSO solvation model for Aprepitant and Ezetimibe. The experimental spectra, the blank spectra and the directory of Gaussian output files were then provided to the *Cai•factor* script with settings of 0.980 for scaling factor and 10 kJ.mol^-1^ as a Boltzmann cut-off value.

We tentatively interpret the improvement in *Cai•factor* at the B3LYP level as reflecting a better match of the calculated Boltzmann population to the conformational population in DMSO solution, and therefore, as some evidence in favour of the rather surprising presence of an 8-membered ring H-bond in DMSO solution.

For the compounds limonene (4) and 2-phenylpropan-1-ol (6), the spectra acquired on the Bruker instrument consistently return near-zero match scores, neither sufficiently positive nor negative to make an assignment. However, spectra given by the BioTools instrument on the same samples return sufficiently positive results for conclusions to be drawn. The following analysis therefore uses the BioTools data for these two compounds. The uncertainty in measurements from the Bruker instrument in this example is interpreted as meaning that a conclusion should not be drawn, which is a better outcome than coming to an incorrect conclusion.

**(a) Scale Factor 0.975**


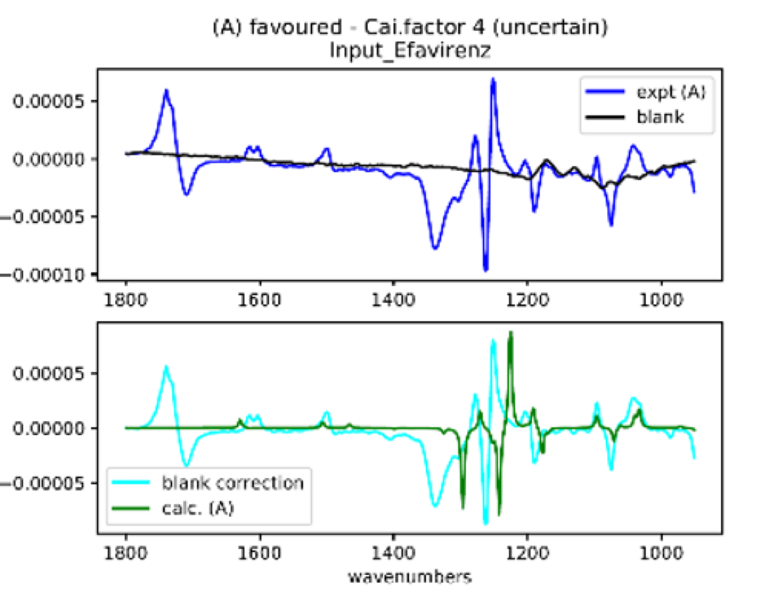


**(b) Scale Factor 0.997**


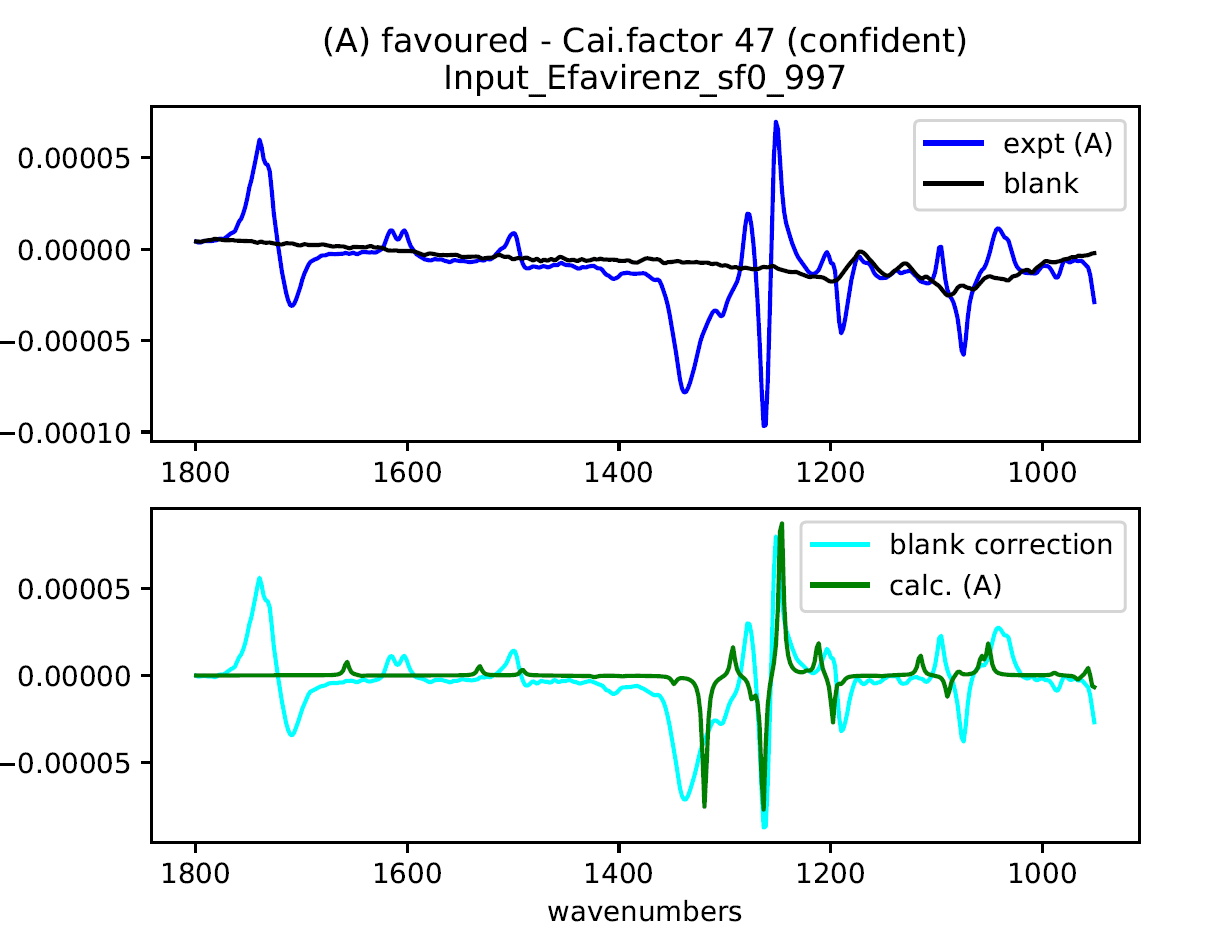


**Fig. S2** Match of experimental and calculated VCD spectra for Efavirenz at a scale factors of (a) 0.975 and (b) 0.997

## Baseline correction

Whilst the average confidence is lower than for taking the spectra of both enantiomers, reliable results are still usually obtained. The results are improved by subtracting the spectrum of the pure solvent from the spectrum of the molecule of interest. In principle, solvents should show no VCD signals, so long as they are achiral, but, in practice, they do not usually generate a completely flat spectrum. Subtracting this baseline from the sample usually gives an improved result.

## Full reference for Gaussian

M. J. Frisch, G. W. Trucks, H. B. Schlegel, G. E. Scuseria, M. A. Robb, J. R. Cheeseman, G. Scalmani, V. Barone, B. Mennucci, G. A. Petersson, H. Nakatsuji, M. Caricato, X. Li, H. P. Hratchian, A. F. Izmaylov, J. Bloino, G. Zheng, J. L. Sonnenberg, M. Hada, M. Ehara, K. Toyota, R. Fukuda, J. Hasegawa, M. Ishida, T. Nakajima, Y. Honda, O. Kitao, H. Nakai, T. Vreven, J. A. Montgomery, J. E. Peralta, F. Ogliaro, M. Bearpark, J. J. Heyd, E. Brothers, K. N. Kudin, V. N. Staroverov, R. Kobayashi, J. Normand, K. Raghavachari, A. Rendell, J. C. Burant, S. S. Iyengar, J. Tomasi, M. Cossi, N. Rega, J. M. Millam, M. Klene, J. E. Knox, J. B. Cross, V. Bakken, C. Adamo, J. Jaramillo, R. Gomperts, R. E. Stratmann, O. Yazyev, A. J. Austin, R. Cammi, C. Pomelli, J. W. Ochterski, R. L. Martin, K. Morokuma, V. G. Zakrzewski, G. A. Voth, P. Salvador, J. J. Dannenberg, S. Dapprich, A. D. Daniels, Ö. Farkas, J. B. Foresman, J. V. Ortiz, J. Cioslowski and D. J. Fox, Gaussian 09, Revision D.01, Gaussian, Inc., Wallingford CT, 2009.

# Molecules and VCD data

The spectra illustrated are all minimized using B3PW91/cc-pVTZ. The upper panel shows the two experimental spectra, and the lower panel compares the combined spectrum with the calculated spectrum

## Molecule 01: epoxybutane

Biotools spectra; neat solution

Bruker spectra; neat solution

## Molecule 02: epichlorohydrin

Biotools spectra; neat solution

Bruker spectra; neat solution

## Molecule 03: but3yn2ol

Biotools spectra; CDCl_3_, 1.0 mol dm^-3^

Bruker spectra; CDCl_3_, 1.0 mol dm^-3^

## Molecule 04: limonene

Biotools spectra; CDCl_3_, 1.0 mol dm^-3^

Bruker spectra; CDCl_3_, 1.0 mol dm^-3^

## Molecule 05: hydroxyfuranone

Biotools spectra; CDCl_3_, 0.8 mol dm^-3^

Bruker spectra; CDCl_3_, 0.8 mol dm^-3^

## Molecule 06: 2phenylpropan1ol

Biotools spectra; CDCl_3_, 0.7 mol dm^-3^

Bruker spectra; CDCl_3_, 0.7 mol dm^-3^

## Molecule 07: chloropropanicacid

Biotools spectra; CDCl_3_, 0.2 mol dm^-3^

Bruker spectra; CDCl_3_, 0.2 mol dm^-3^

## Molecule 08: AnilHex

Biotools spectra; CDCl_3_, 0.5 mol dm^-3^

## Molecule 09: camphor

Biotools spectra; CDCl_3_, 1.0 mol dm^-3^

## Molecule 10: ImBAiso

Biotools spectra; CDCl_3_, 1.0 mol dm^-3^

## Molecule 11: arylpip

Biotools spectra; CDCl_3_, 1.0 mol dm^-3^

## Molecule 12: 2isopropyl: pyrazine

Biotools spectra; CDCl_3_, 1.0 mol dm^-3^

## Molecule 13: PropONO

Biotools spectra; CDCl_3_, 1.0 mol dm^-3^

## Molecule 14: naphethylamine

Biotools spectra; CDCl_3_, 1.0 mol dm^-3^

## Molecule 15: MCPOPCx

Biotools spectra; CDCl_3_, 1.0 mol dm^-3^

## Molecule 16: BayerCp

Biotools spectra; CDCl_3_, 1.0 mol dm^-3^

## Molecule 17: MeOPhAA

Biotools spectra; CDCl_3_, 0.9 mol dm^-3^

## Molecule 18: ClThaln

Bruker spectra; DMSO, 0.9 mol dm^-3^. Note strong solvent signal below 1100 cm^-1^ so a range of 1100 cm^-1^ – 1600 cm^-1^ is clearer.

## Molecule 19: cPrEtAm

Biotools spectra; CDCl_3_, 1.0 mol dm^-3^

## Molecule 20: 4hydroxypyrrolidinone

Biotools spectra; CDCl_3_, 0.8 mol dm^-3^

## Molecule 21: MeOTFPA

Biotools spectra; CDCl_3_, 0.9 mol dm^-3^

## Molecule 22: BrFOpyr

Biotools spectra; DMSO, 1.0 mol dm^-3^

## Molecule 23: MForMor

Biotools spectra; CDCl_3_, 1.0 mol dm^-3^

## Molecule 24: sparteine

Bruker spectra; CDCl_3_, 0.4 mol dm^-3^

## Molecule 25: Me2PEAm

Bruker spectra; CDCl_3_, 0.9 mol dm^-3^

## Molecule 26: ClPPrOH

Bruker spectra; CDCl_3_, 1.2 mol dm^-3^

## Molecule 27: LacMide

Bruker spectra; CDCl_3_, 1.2 mol dm^-3^

## Molecule 28: ClHBuCN

Bruker spectra; CDCl_3_, 1.3 mol dm^-3^

## Molecule 29: MePhEAm

Bruker spectra; CDCl_3_, 0.9 mol dm^-3^

## Molecule 30: TBPTA00

Bruker spectra; CDCl_3_, 0.4 mol dm^-3^

## Aprepitant

**Input file**

<Settings>

title: Aprepitant RRS

broadening: 5.0

minimum_wavenumber: 1100.0

maximum_wavenumber: 1800.0

temperature: 298.0

defined_scaling_factor: 0.98

boltzmann_cutoff: 10.0

print_csv spectra scaling_factor summary

<Experiments>

../Experimental/Aprepitant_dilute_VCD_spectrum_averaged.csv

<Blank>

../Experimental/DMSO_blank_VCD_spectrum_averaged.csv

<Calculations>

../Calculation/RRS

**Output file**

##########################################

## Cai•VCD analysis ##

## University of Cambridge, 2020 ##

##########################################

Input_Aprepitant_RRS_new_search

minimum wavenumber: 1100.0

maximum wavenumber: 1800.0

Lorentzian broadening: 5.0

Temperature for Boltzmann averaging: 298.0 K

Defined scaling factor: 0.98

Print out graphs: True

Boltzmann Analysis: True

Boltzmann Analysis Energy cut-off: 10.0

Unique calculated structure criteria: Energy: 0.0001 Frequency: 2.0

Extreme scale factor warning range: 0.01

Insufficient information criterion for Cai.factor: 10

Printing spectra in .csv file

Printing scaling factor analysis in .csv file

Printing two-line summary in .csv file

Experimental data for single enantiomer:

(A) ../Experimental/Aprepitant_dilute_VCD_spectrum_averaged.csv

Blank file:

../Experimental/DMSO_blank_VCD_spectrum_averaged.csv

Calculation files:

../Calculation/RRS_new_search

Only one filename listed for calculations

which is a directory

Number of files: 68

Calculated data in Gaussian file

50 files rejected by energy cutoff

6 duplicate files removed

Unique Calculated Structures

Energy: -2032.300631 hartrees, 0.000 kJ/mol, Boltzmann Factor: 1.000 ../Calculation/RRS_new_search/conf_1_B3PW91_cc-pVTZ

Energy: -2032.300446 hartrees, 0.486 kJ/mol, Boltzmann Factor: 0.822 ../Calculation/RRS_new_search/conf_3_B3PW91_cc-pVTZ

Energy: -2032.299669 hartrees, 2.526 kJ/mol, Boltzmann Factor: 0.361 ../Calculation/RRS_new_search/conf_18_B3PW91_cc-pVTZ

Energy: -2032.298694 hartrees, 5.086 kJ/mol, Boltzmann Factor: 0.128 ../Calculation/RRS_new_search/conf_35_B3PW91_cc-pVTZ

Energy: -2032.298505 hartrees, 5.582 kJ/mol, Boltzmann Factor: 0.105 ../Calculation/RRS_new_search/conf_57_B3PW91_cc-pVTZ

Energy: -2032.298291 hartrees, 6.144 kJ/mol, Boltzmann Factor: 0.084 ../Calculation/RRS_new_search/conf_20_B3PW91_cc-pVTZ

Energy: -2032.298290 hartrees, 6.147 kJ/mol, Boltzmann Factor: 0.084 ../Calculation/RRS_new_search/conf_58_B3PW91_cc-pVTZ

Energy: -2032.297804 hartrees, 7.423 kJ/mol, Boltzmann Factor: 0.050 ../Calculation/RRS_new_search/conf_5_B3PW91_cc-pVTZ

Energy: -2032.297354 hartrees, 8.605 kJ/mol, Boltzmann Factor: 0.031 ../Calculation/RRS_new_search/conf_11_B3PW91_cc-pVTZ

Energy: -2032.297349 hartrees, 8.618 kJ/mol, Boltzmann Factor: 0.031 ../Calculation/RRS_new_search/conf_33_B3PW91_cc-pVTZ

Energy: -2032.297107 hartrees, 9.253 kJ/mol, Boltzmann Factor: 0.024 ../Calculation/RRS_new_search/conf_34_B3PW91_cc-pVTZ

Energy: -2032.297011 hartrees, 9.505 kJ/mol, Boltzmann Factor: 0.022 ../Calculation/RRS_new_search/conf_54_B3PW91_cc-pVTZ

Using all 12 unique conformations within energy cut-off

Defined scaling factor: 0.98

Single enantiomer result: Defined SF : 0.980 ; File (A) is assigned to the enantiomer calculated with Cai.factor 26

Single enantiomer blank: Defined SF : 0.980 ; File (A) is assigned to the enantiomer calculated with Cai.factor 28

Single enantiomer result: Opt. SF : 0.984 ; File (A) is assigned to the enantiomer calculated with Cai.factor 34

Single enantiomer blank: Opt. SF : 0.984 ; File (A) is assigned to the enantiomer calculated with Cai.factor 40

Experimental data for single enantiomer:

(A) ../Experimental/Aprepitant_dilute_VCD_spectrum_averaged.csv

Blank file:

../Experimental/DMSO_blank_VCD_spectrum_averaged.csv

Single enantiomer summary

This is based on File (A) ( cautiously confident )

Using the blank data, File (A) is cautiously confident

Optimising the scale factor increases the confidence level: fairly confident

Overall Cai.factor is 35 which means fairly confident assignment


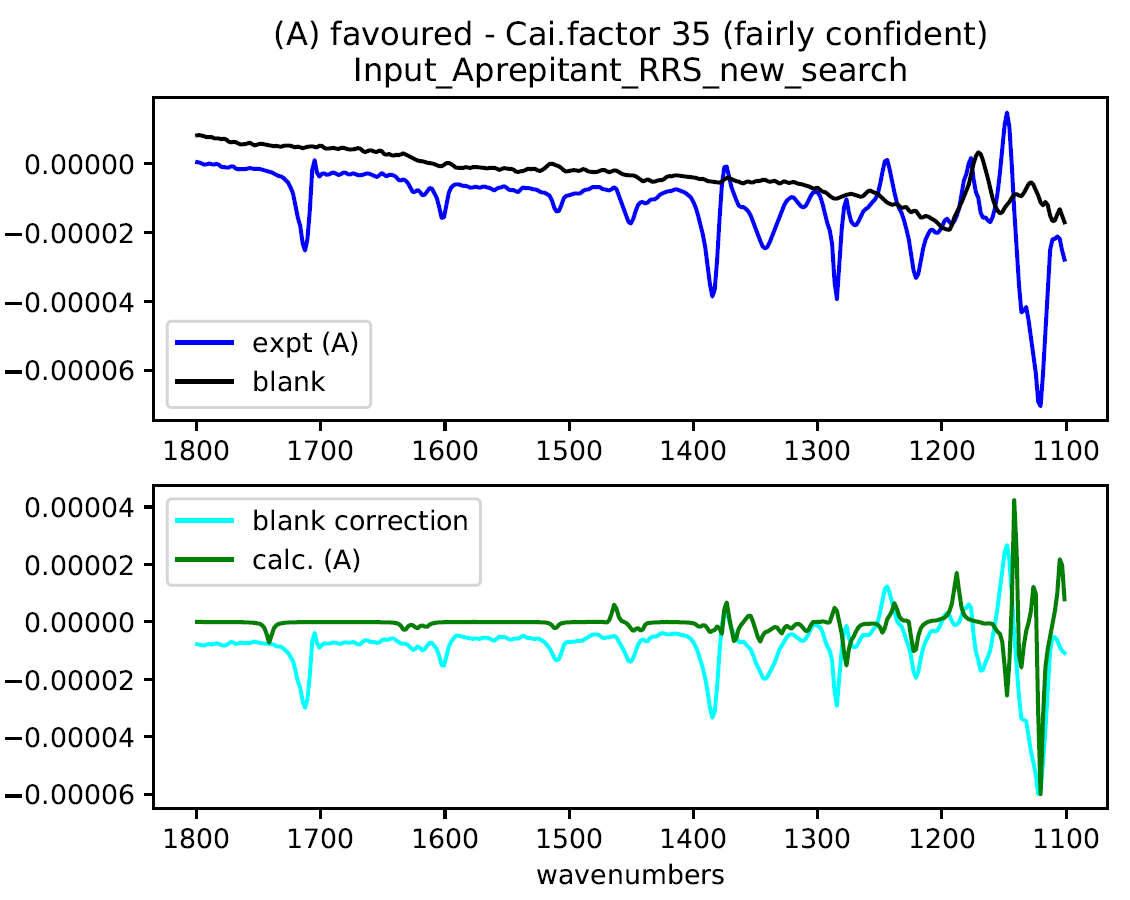


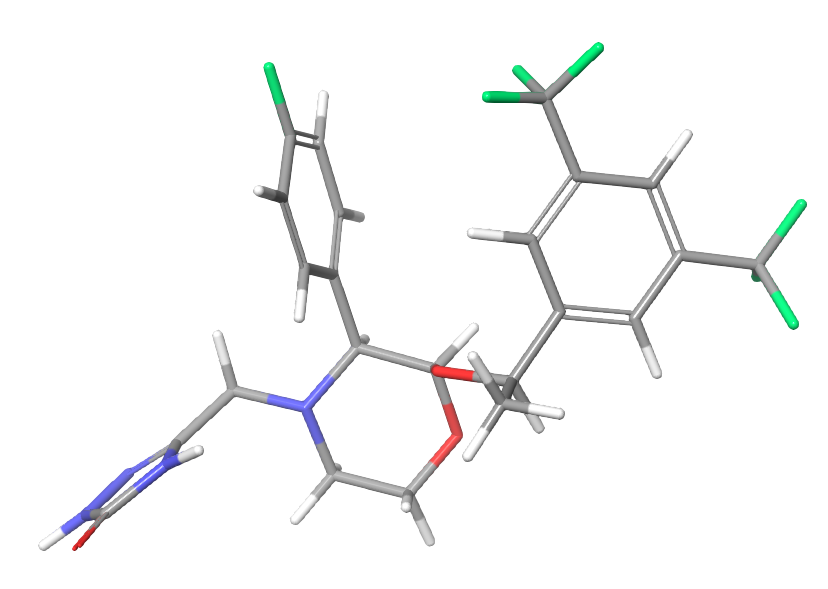


Conf 1 (minimum in Boltzmann)

O -0.77300 -2.59750 -1.04740

C -0.54200 -1.25150 -0.72270

C -1.68720 -0.34390 -1.21670

N -2.97840 -0.82990 -0.71800

C -3.15530 -2.23850 -1.06750

C -2.01690 -3.07010 -0.53470

H 0.36790 -0.98220 -1.26820

O -0.35870 -1.07030 0.65720

H -1.65130 -0.44180 -2.31500

C -1.42350 1.10600 -0.88470

H -4.09420 -2.59800 -0.64180

H -3.21800 -2.36350 -2.16170

H -2.11030 -4.10350 -0.86550

H -2.00530 -3.05050 0.55860

C -4.09120 -0.04210 -1.23330

C -5.35350 -0.33240 -0.50190

H -3.85320 1.01620 -1.10870

H -4.27870 -0.21140 -2.30400

N -6.50850 -0.52750 -1.05760

N -5.44740 -0.37700 0.86070

N -7.36860 -0.69300 -0.00610

H -8.34300 -0.87470 -0.16940

C -6.76340 -0.62180 1.21670

H -4.68460 -0.29630 1.51080

O -7.24540 -0.74050 2.33250

C -1.65070 1.62450 0.39040

C -0.91840 1.95360 -1.86690

C -1.38500 2.95400 0.67920

C -0.63570 3.28660 -1.59600

C -0.87760 3.76120 -0.32300

H -0.74460 1.57210 -2.86640

H -1.56270 3.36390 1.66480

H -2.03960 0.97870 1.16510

F -0.61560 5.05400 -0.04680

H -0.24270 3.94730 -2.35710

C 0.78750 -1.72720 1.19840

C 2.07990 -1.06000 0.77200

C 0.62540 -1.71930 2.70840

H 0.80490 -2.76130 0.84350

C 2.21060 0.32600 0.79710

C 3.17140 -1.83020 0.39540

C 3.41690 0.92100 0.46040

C 4.37940 -1.22840 0.06000

C 4.51080 0.14960 0.08780

H 3.08180 -2.90940 0.36910

C 3.53980 2.41880 0.45520

H 1.36370 0.93670 1.08120

H 5.45030 0.61710 -0.16980

C 5.52880 -2.09190 -0.37690

F 3.25410 2.93710 -0.75750

F 2.70450 3.00120 1.32990

F 4.78300 2.82530 0.76250

F 5.64690 -3.19330 0.38580

F 6.70360 -1.44670 -0.32670

F 5.37250 -2.52060 -1.64630

H 1.46470 -2.22930 3.18170

H 0.58650 -0.69610 3.08530

H -0.29590 -2.23140 2.98720

## Efavirenz

**Input file (scale factor 0.98)**

<Settings>

title: Efavirenz

broadening: 5.0

minimum_wavenumber: 950.0

maximum_wavenumber: 1800.0

temperature: 298.0

defined_scaling_factor: 0.98

boltzmann_cutoff: 10.0

print_csv spectra scaling_factor summary

<Experiments>

../Experimental/Efavirenz_VCD_spectrum_averaged.csv

<Blank>

../Experimental/CDCl3_blank_VCD_spectrum_averaged.csv

<Calculations>

../Calculation/S

**Output file (scale factor 0.98)**

##########################################

## Cai•VCD analysis ##

## University of Cambridge, 2020 ##

##########################################

Input_Efavirenz

minimum wavenumber: 950.0

maximum wavenumber: 1800.0

Lorentzian broadening: 5.0

Temperature for Boltzmann averaging: 298.0 K

Defined scaling factor: 0.98

Print out graphs: True

Boltzmann Analysis: True

Boltzmann Analysis Energy cut-off: 10.0

Unique calculated structure criteria: Energy: 0.0001 Frequency: 2.0

Extreme scale factor warning range: 0.01

Insufficient information criterion for Cai.factor: 10

Printing spectra in .csv file

Printing scaling factor analysis in .csv file

Printing two-line summary in .csv file

Experimental data for single enantiomer:

(A) ../Experimental/Efavirenz_VCD_spectrum_averaged.csv

Blank file:

../Experimental/CDCl3_blank_VCD_spectrum_averaged.csv

Calculation files:

../Calculation/S

Only one filename listed for calculations

which is a directory

Number of files: 10

Calculated data in Gaussian file

0 files rejected by energy cutoff

8 duplicate files removed

Unique Calculated Structures

Energy: -1503.566233 hartrees, 0.000 kJ/mol, Boltzmann Factor: 1.000 ../Calculation/S/conf_5_B3PW91_cc-pVTZ

Energy: -1503.565791 hartrees, 1.161 kJ/mol, Boltzmann Factor: 0.626 ../Calculation/S/conf_1_B3PW91_cc-pVTZ

Using all 2 unique conformations within energy cut-off

Defined scaling factor: 0.98

Single enantiomer result: Defined SF : 0.980 ; Insufficient information for a definite conclusion (B) with Cai.factor 1

Single enantiomer blank: Defined SF : 0.980 ; Insufficient information for a definite conclusion (A) with Cai.factor 1

Single enantiomer result: Opt. SF : 0.997 ; File (A) is assigned to the enantiomer calculated with Cai.factor 41

Single enantiomer blank: Opt. SF : 0.997 ; File (A) is assigned to the enantiomer calculated with Cai.factor 47

Experimental data for single enantiomer:

(A) ../Experimental/Efavirenz_VCD_spectrum_averaged.csv

Blank file:

../Experimental/CDCl3_blank_VCD_spectrum_averaged.csv

Single enantiomer summary

This is based on File (A) ( uncertain )

Using the blank data, File (A) is uncertain

Optimising the scale factor increases the confidence level: uncertain

Overall Cai.factor is 4 which means uncertain assignment


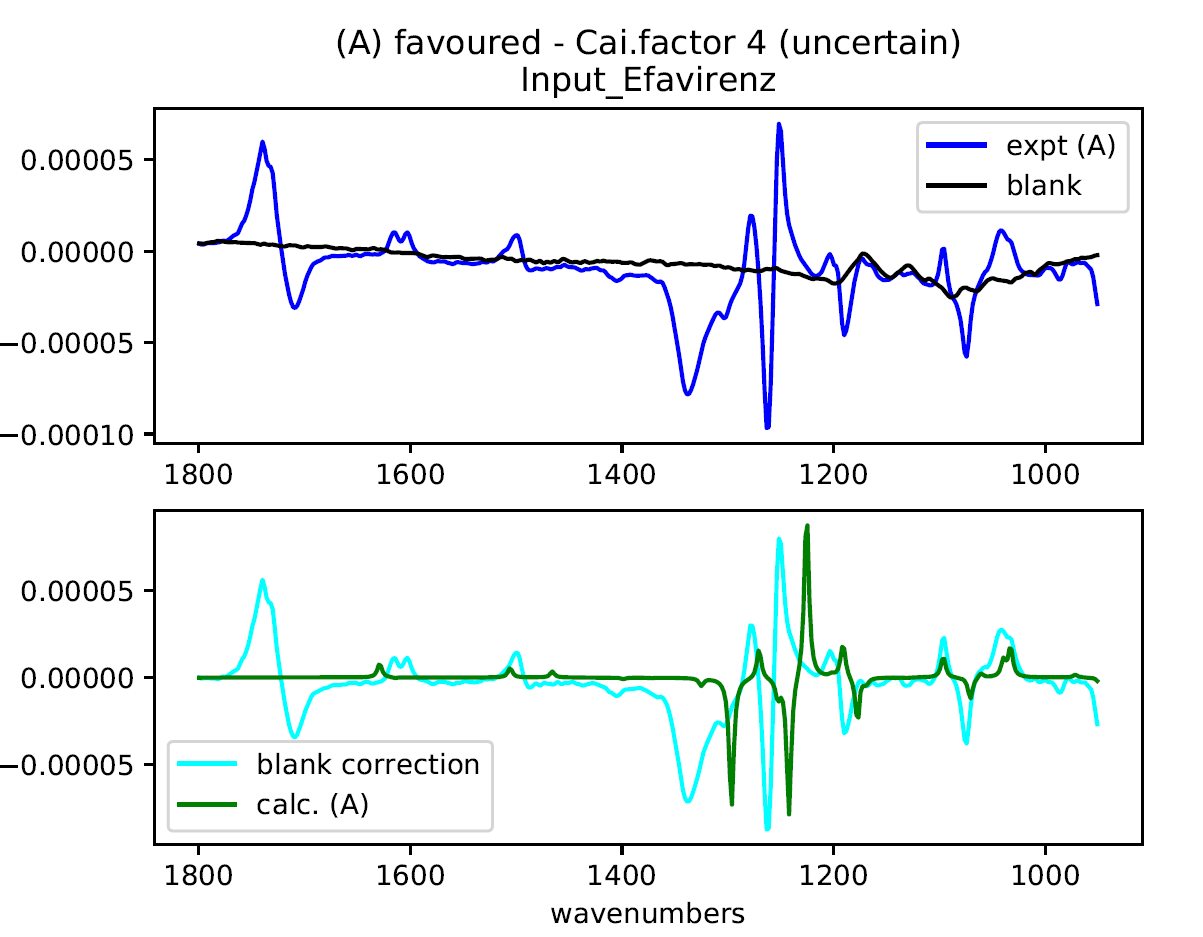


**Input file (scale factor 0.98)**

<Settings>

title: Efavirenz

broadening: 5.0

minimum_wavenumber: 950.0

maximum_wavenumber: 1800.0

temperature: 298.0

defined_scaling_factor: 0.997

boltzmann_cutoff: 10.0

print_csv spectra scaling_factor summary

<Experiments>

../Experimental/Efavirenz_VCD_spectrum_averaged.csv

<Blank>

../Experimental/CDCl3_blank_VCD_spectrum_averaged.csv

<Calculations>

../Calculation/S

**Output file (scale factor 0.98)**

##########################################

## Cai•VCD analysis ##

## University of Cambridge, 2020 ##

##########################################

Input_Efavirenz_sf0_997

minimum wavenumber: 950.0

maximum wavenumber: 1800.0

Lorentzian broadening: 5.0

Temperature for Boltzmann averaging: 298.0 K

Defined scaling factor: 0.997

Print out graphs: True

Boltzmann Analysis: True

Boltzmann Analysis Energy cut-off: 10.0

Unique calculated structure criteria: Energy: 0.0001 Frequency: 2.0

Extreme scale factor warning range: 0.01

Insufficient information criterion for Cai.factor: 10

Printing spectra in .csv file

Printing scaling factor analysis in .csv file

Printing two-line summary in .csv file

Experimental data for single enantiomer:

(A) ../Experimental/Efavirenz_VCD_spectrum_averaged.csv

Blank file:

../Experimental/CDCl3_blank_VCD_spectrum_averaged.csv

Calculation files:

../Calculation/S

Only one filename listed for calculations

which is a directory

Number of files: 10

Calculated data in Gaussian file

0 files rejected by energy cutoff

8 duplicate files removed

Unique Calculated Structures

Energy: -1503.566233 hartrees, 0.000 kJ/mol, Boltzmann Factor: 1.000 ../Calculation/S/conf_5_B3PW91_cc-pVTZ

Energy: -1503.565791 hartrees, 1.161 kJ/mol, Boltzmann Factor: 0.626 ../Calculation/S/conf_1_B3PW91_cc-pVTZ

Using all 2 unique conformations within energy cut-off

Defined scaling factor: 0.997

Single enantiomer result: Defined SF : 0.997 ; File (A) is assigned to the enantiomer calculated with Cai.factor 41

Single enantiomer blank: Defined SF : 0.997 ; File (A) is assigned to the enantiomer calculated with Cai.factor 47

Single enantiomer result: Opt. SF : 0.997 ; File (A) is assigned to the enantiomer calculated with Cai.factor 41

Single enantiomer blank: Opt. SF : 0.997 ; File (A) is assigned to the enantiomer calculated with Cai.factor 47

Experimental data for single enantiomer:

(A) ../Experimental/Efavirenz_VCD_spectrum_averaged.csv

Blank file:

../Experimental/CDCl3_blank_VCD_spectrum_averaged.csv

Single enantiomer summary

This is based on File (A) ( confident )

Using the blank data, File (A) is confident

Optimising the scale factor does not improve overall confidence substantially

Overall Cai.factor is 47 which means confident assignment


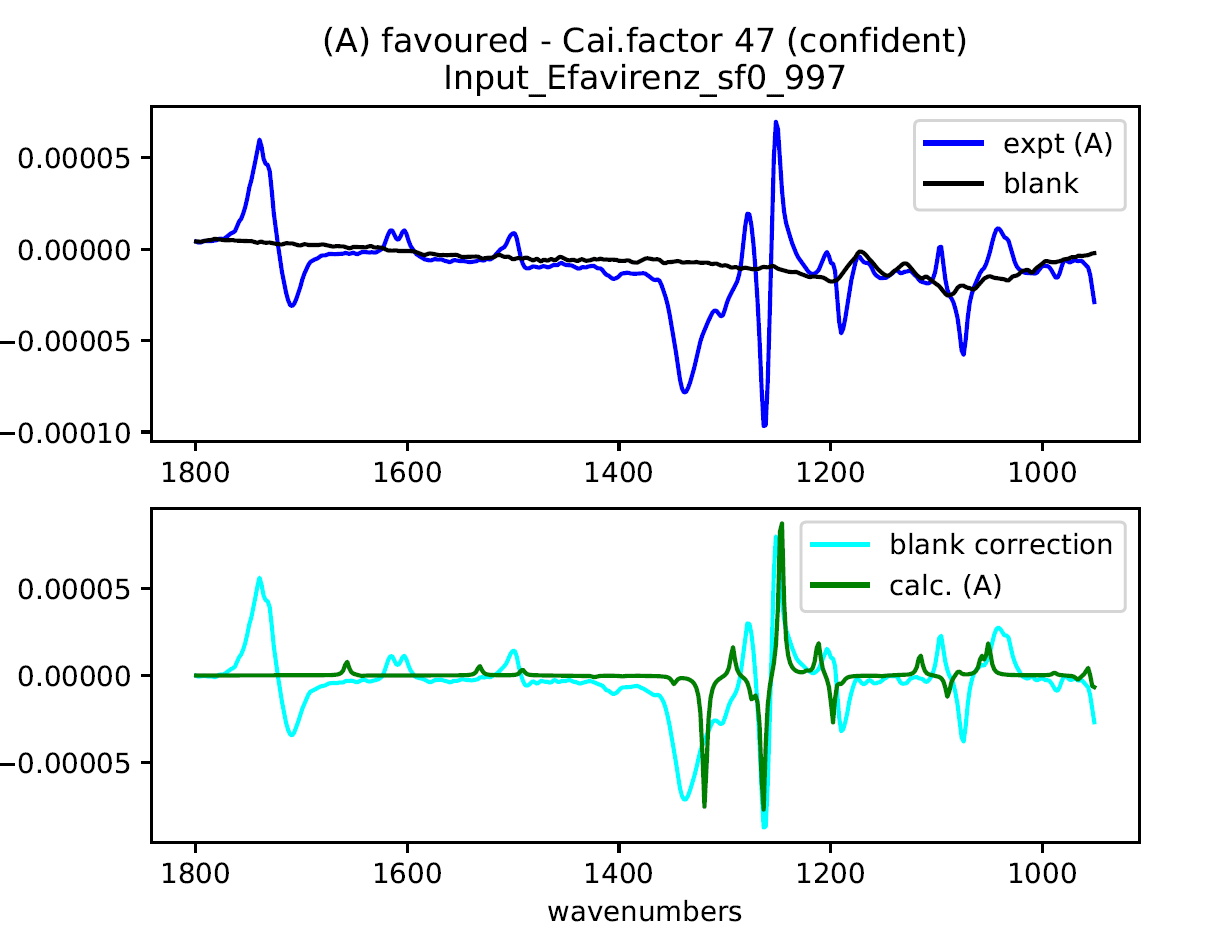


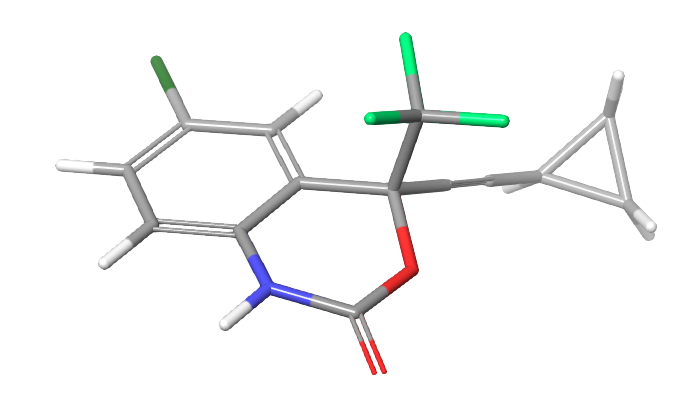


Conf 5 (Boltzmann minimum)

C -1.95440 -0.91180 -0.59710

C -0.92750 -0.07300 -0.17420

C -1.18180 1.26920 0.06010

C -2.46470 1.76400 -0.10360

C -3.49750 0.93150 -0.51040

C -3.23960 -0.40360 -0.76370

N -1.66470 -2.24170 -0.85670

C 0.44250 -0.68490 0.04040

H -0.38010 1.92310 0.37340

Cl -2.78200 3.44480 0.20080

H -4.49480 1.32970 -0.63780

H -4.03840 -1.05450 -1.09820

C 0.62670 -1.08460 1.53840

O 0.61730 -1.89960 -0.71490

C 1.51520 0.21970 -0.33720

H -2.39890 -2.86900 -1.13880

C -0.40180 -2.75100 -0.99820

O -0.17430 -3.86170 -1.38620

F 0.52670 -0.01380 2.33140

F -0.31590 -1.96110 1.91050

F 1.81350 -1.64850 1.74660

C 2.40490 0.96930 -0.64220

C 3.47040 1.84800 -1.00360

H 3.18510 2.66720 -1.65270

C 4.57850 2.13370 -0.01120

C 4.86410 1.29020 -1.20440

H 4.95850 3.14590 0.01300

H 4.50940 1.64690 0.95130

H 5.44280 1.71350 -2.01390

H 4.98900 0.22670 -1.05870

## Ezetimibe

1. *ab initio* calculations at B3PW91 / cc-pVTZ

**Input file**

<Settings>

title: Ezetimibe SRS

broadening: 5.0

minimum_wavenumber: 1100.0

maximum_wavenumber: 1800.0

temperature: 298.0

defined_scaling_factor: 0.98

boltzmann_cutoff: 10.0

print_csv spectra scaling_factor summary

<Experiments>

../../Experimental_DMSO/Ezetimibe_VCD_spectrum_averaged.csv

<Blank>

../../Experimental_DMSO/DMSO_blank_VCD_spectrum_averaged.csv

<Calculations>

../../Calculation_DMSO/SRS_new_search

**Output file**

##########################################

## Cai•VCD analysis ##

## University of Cambridge, 2020 ##

##########################################

Input_Ezetimibe_SRS_new_search

minimum wavenumber: 1100.0

maximum wavenumber: 1800.0

Lorentzian broadening: 5.0

Temperature for Boltzmann averaging: 298.0 K

Defined scaling factor: 0.98

Print out graphs: True

Boltzmann Analysis: True

Boltzmann Analysis Energy cut-off: 10.0

Unique calculated structure criteria: Energy: 0.0001 Frequency: 2.0

Extreme scale factor warning range: 0.01

Insufficient information criterion for Cai.factor: 10

Printing spectra in .csv file

Printing scaling factor analysis in .csv file

Printing two-line summary in .csv file

Experimental data for single enantiomer:

(A) ../../Experimental_DMSO/Ezetimibe_VCD_spectrum_averaged.csv

Blank file:

../../Experimental_DMSO/DMSO_blank_VCD_spectrum_averaged.csv

Calculation files:

../../Calculation_DMSO/SRS_new_search

Only one filename listed for calculations

which is a directory

Number of files: 130

Calculated data in Gaussian file

71 files rejected by energy cutoff

10 duplicate files removed

Unique Calculated Structures

Energy: -1406.935315 hartrees, 0.000 kJ/mol, Boltzmann Factor: 1.000 ../../Calculation_DMSO/SRS_new_search/conf2_10_B3PW91_cc-pVTZ

Energy: -1406.935021 hartrees, 0.772 kJ/mol, Boltzmann Factor: 0.732 ../../Calculation_DMSO/SRS_new_search/conf2_18_B3PW91_cc-pVTZ

Energy: -1406.934702 hartrees, 1.610 kJ/mol, Boltzmann Factor: 0.522 ../../Calculation_DMSO/SRS_new_search/conf2_56_B3PW91_cc-pVTZ

Energy: -1406.934609 hartrees, 1.854 kJ/mol, Boltzmann Factor: 0.473 ../../Calculation_DMSO/SRS_new_search/conf2_75_B3PW91_cc-pVTZ

Energy: -1406.934076 hartrees, 3.253 kJ/mol, Boltzmann Factor: 0.269 ../../Calculation_DMSO/SRS_new_search/conf2_59_B3PW91_cc-pVTZ

Energy: -1406.933984 hartrees, 3.495 kJ/mol, Boltzmann Factor: 0.244 ../../Calculation_DMSO/SRS_new_search/conf2_39_B3PW91_cc-pVTZ

Energy: -1406.933837 hartrees, 3.881 kJ/mol, Boltzmann Factor: 0.209 ../../Calculation_DMSO/SRS_new_search/conf2_45_B3PW91_cc-pVTZ

Energy: -1406.933779 hartrees, 4.033 kJ/mol, Boltzmann Factor: 0.196 ../../Calculation_DMSO/SRS_new_search/conf2_16_B3PW91_cc-pVTZ

Energy: -1406.933670 hartrees, 4.319 kJ/mol, Boltzmann Factor: 0.175 ../../Calculation_DMSO/SRS_new_search/conf2_57_B3PW91_cc-pVTZ

Energy: -1406.933661 hartrees, 4.343 kJ/mol, Boltzmann Factor: 0.173 ../../Calculation_DMSO/SRS_new_search/conf2_63_B3PW91_cc-pVTZ

Energy: -1406.933602 hartrees, 4.498 kJ/mol, Boltzmann Factor: 0.163 ../../Calculation_DMSO/SRS_new_search/conf2_31_B3PW91_cc-pVTZ

Energy: -1406.933569 hartrees, 4.585 kJ/mol, Boltzmann Factor: 0.157 ../../Calculation_DMSO/SRS_new_search/conf2_87_B3PW91_cc-pVTZ

Energy: -1406.933539 hartrees, 4.663 kJ/mol, Boltzmann Factor: 0.152 ../../Calculation_DMSO/SRS_new_search/conf2_49_B3PW91_cc-pVTZ

Energy: -1406.933531 hartrees, 4.684 kJ/mol, Boltzmann Factor: 0.151 ../../Calculation_DMSO/SRS_new_search/conf2_78_B3PW91_cc-pVTZ

Energy: -1406.933442 hartrees, 4.918 kJ/mol, Boltzmann Factor: 0.137 ../../Calculation_DMSO/SRS_new_search/conf2_5_B3PW91_cc-pVTZ

Energy: -1406.933440 hartrees, 4.923 kJ/mol, Boltzmann Factor: 0.137 ../../Calculation_DMSO/SRS_new_search/conf2_62_B3PW91_cc-pVTZ

Energy: -1406.933337 hartrees, 5.194 kJ/mol, Boltzmann Factor: 0.123 ../../Calculation_DMSO/SRS_new_search/conf2_104_B3PW91_cc-pVTZ

Energy: -1406.933275 hartrees, 5.357 kJ/mol, Boltzmann Factor: 0.115 ../../Calculation_DMSO/SRS_new_search/conf2_88_B3PW91_cc-pVTZ

Energy: -1406.933256 hartrees, 5.407 kJ/mol, Boltzmann Factor: 0.113 ../../Calculation_DMSO/SRS_new_search/conf2_79_B3PW91_cc-pVTZ

Energy: -1406.933164 hartrees, 5.648 kJ/mol, Boltzmann Factor: 0.102 ../../Calculation_DMSO/SRS_new_search/conf2_71_B3PW91_cc-pVTZ

Energy: -1406.933155 hartrees, 5.672 kJ/mol, Boltzmann Factor: 0.101 ../../Calculation_DMSO/SRS_new_search/conf2_68_B3PW91_cc-pVTZ

Energy: -1406.932903 hartrees, 6.333 kJ/mol, Boltzmann Factor: 0.078 ../../Calculation_DMSO/SRS_new_search/conf2_77_B3PW91_cc-pVTZ

Energy: -1406.932882 hartrees, 6.389 kJ/mol, Boltzmann Factor: 0.076 ../../Calculation_DMSO/SRS_new_search/conf2_55_B3PW91_cc-pVTZ

Energy: -1406.932851 hartrees, 6.470 kJ/mol, Boltzmann Factor: 0.073 ../../Calculation_DMSO/SRS_new_search/conf2_106_B3PW91_cc-pVTZ

Energy: -1406.932815 hartrees, 6.565 kJ/mol, Boltzmann Factor: 0.071 ../../Calculation_DMSO/SRS_new_search/conf2_93_B3PW91_cc-pVTZ

Energy: -1406.932694 hartrees, 6.882 kJ/mol, Boltzmann Factor: 0.062 ../../Calculation_DMSO/SRS_new_search/conf2_115_B3PW91_cc-pVTZ

Energy: -1406.932494 hartrees, 7.407 kJ/mol, Boltzmann Factor: 0.050 ../../Calculation_DMSO/SRS_new_search/conf2_64_B3PW91_cc-pVTZ

Energy: -1406.932484 hartrees, 7.434 kJ/mol, Boltzmann Factor: 0.050 ../../Calculation_DMSO/SRS_new_search/conf2_82_B3PW91_cc-pVTZ

Energy: -1406.932461 hartrees, 7.494 kJ/mol, Boltzmann Factor: 0.049 ../../Calculation_DMSO/SRS_new_search/conf2_43_B3PW91_cc-pVTZ

Energy: -1406.932446 hartrees, 7.533 kJ/mol, Boltzmann Factor: 0.048 ../../Calculation_DMSO/SRS_new_search/conf2_33_B3PW91_cc-pVTZ

Energy: -1406.932414 hartrees, 7.617 kJ/mol, Boltzmann Factor: 0.046 ../../Calculation_DMSO/SRS_new_search/conf2_19_B3PW91_cc-pVTZ

Energy: -1406.932413 hartrees, 7.620 kJ/mol, Boltzmann Factor: 0.046 ../../Calculation_DMSO/SRS_new_search/conf2_36_B3PW91_cc-pVTZ

Energy: -1406.932392 hartrees, 7.675 kJ/mol, Boltzmann Factor: 0.045 ../../Calculation_DMSO/SRS_new_search/conf2_25_B3PW91_cc-pVTZ

Energy: -1406.932356 hartrees, 7.770 kJ/mol, Boltzmann Factor: 0.043 ../../Calculation_DMSO/SRS_new_search/conf2_76_B3PW91_cc-pVTZ

Energy: -1406.932322 hartrees, 7.859 kJ/mol, Boltzmann Factor: 0.042 ../../Calculation_DMSO/SRS_new_search/conf2_50_B3PW91_cc-pVTZ

Energy: -1406.932299 hartrees, 7.919 kJ/mol, Boltzmann Factor: 0.041 ../../Calculation_DMSO/SRS_new_search/conf2_14_B3PW91_cc-pVTZ

Energy: -1406.932281 hartrees, 7.967 kJ/mol, Boltzmann Factor: 0.040 ../../Calculation_DMSO/SRS_new_search/conf2_117_B3PW91_cc-pVTZ

Energy: -1406.932136 hartrees, 8.347 kJ/mol, Boltzmann Factor: 0.034 ../../Calculation_DMSO/SRS_new_search/conf2_110_B3PW91_cc-pVTZ

Energy: -1406.932002 hartrees, 8.699 kJ/mol, Boltzmann Factor: 0.030 ../../Calculation_DMSO/SRS_new_search/conf2_95_B3PW91_cc-pVTZ

Energy: -1406.931958 hartrees, 8.815 kJ/mol, Boltzmann Factor: 0.029 ../../Calculation_DMSO/SRS_new_search/conf2_65_B3PW91_cc-pVTZ

Energy: -1406.931955 hartrees, 8.823 kJ/mol, Boltzmann Factor: 0.028 ../../Calculation_DMSO/SRS_new_search/conf2_94_B3PW91_cc-pVTZ

Energy: -1406.931953 hartrees, 8.828 kJ/mol, Boltzmann Factor: 0.028 ../../Calculation_DMSO/SRS_new_search/conf2_58_B3PW91_cc-pVTZ

Energy: -1406.931922 hartrees, 8.909 kJ/mol, Boltzmann Factor: 0.027 ../../Calculation_DMSO/SRS_new_search/conf2_53_B3PW91_cc-pVTZ

Energy: -1406.931879 hartrees, 9.022 kJ/mol, Boltzmann Factor: 0.026 ../../Calculation_DMSO/SRS_new_search/conf2_91_B3PW91_cc-pVTZ

Energy: -1406.931873 hartrees, 9.038 kJ/mol, Boltzmann Factor: 0.026 ../../Calculation_DMSO/SRS_new_search/conf2_80_B3PW91_cc-pVTZ

Energy: -1406.931837 hartrees, 9.133 kJ/mol, Boltzmann Factor: 0.025 ../../Calculation_DMSO/SRS_new_search/conf2_103_B3PW91_cc-pVTZ

Energy: -1406.931755 hartrees, 9.348 kJ/mol, Boltzmann Factor: 0.023 ../../Calculation_DMSO/SRS_new_search/conf2_112_B3PW91_cc-pVTZ

Energy: -1406.931602 hartrees, 9.750 kJ/mol, Boltzmann Factor: 0.020 ../../Calculation_DMSO/SRS_new_search/conf2_118_B3PW91_cc-pVTZ

Energy: -1406.931543 hartrees, 9.905 kJ/mol, Boltzmann Factor: 0.018 ../../Calculation_DMSO/SRS_new_search/conf2_114_B3PW91_cc-pVTZ

Using all 49 unique conformations within energy cut-off

Defined scaling factor: 0.98

Single enantiomer result: Defined SF : 0.980 ; File (A) is assigned to the enantiomer calculated with Cai.factor 16

Single enantiomer blank: Defined SF : 0.980 ; File (A) is assigned to the enantiomer calculated with Cai.factor 16

Single enantiomer result: Opt. SF : 0.977 ; File (A) is assigned to the enantiomer calculated with Cai.factor 21

Single enantiomer blank: Opt. SF : 0.977 ; File (A) is assigned to the enantiomer calculated with Cai.factor 23

Experimental data for single enantiomer:

(A) ../../Experimental_DMSO/Ezetimibe_VCD_spectrum_averaged.csv

Blank file:

../../Experimental_DMSO/DMSO_blank_VCD_spectrum_averaged.csv

Single enantiomer summary

This is based on File (A) ( possible )

Using the blank data, File (A) is possible

Optimising the scale factor increases the confidence level: cautiously confident

Overall Cai.factor is 21 which means cautiously confident assignment


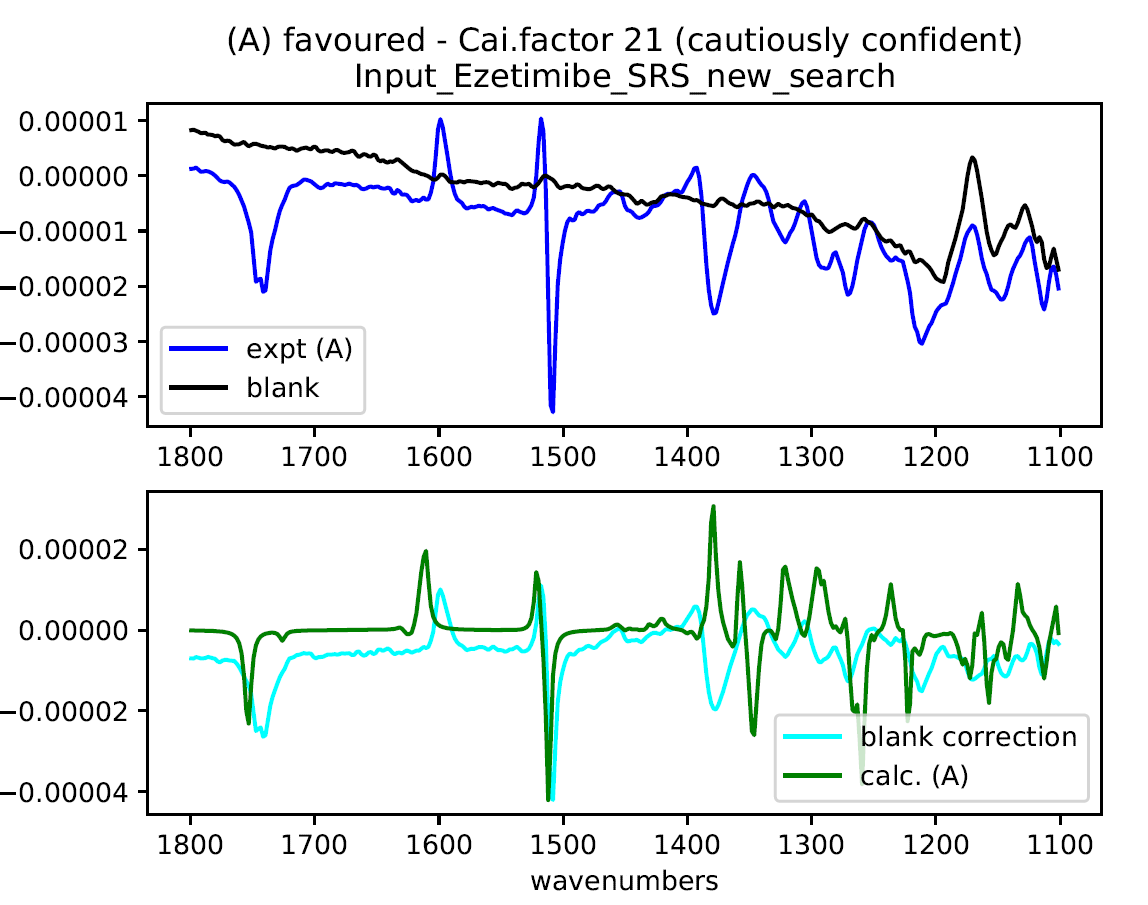


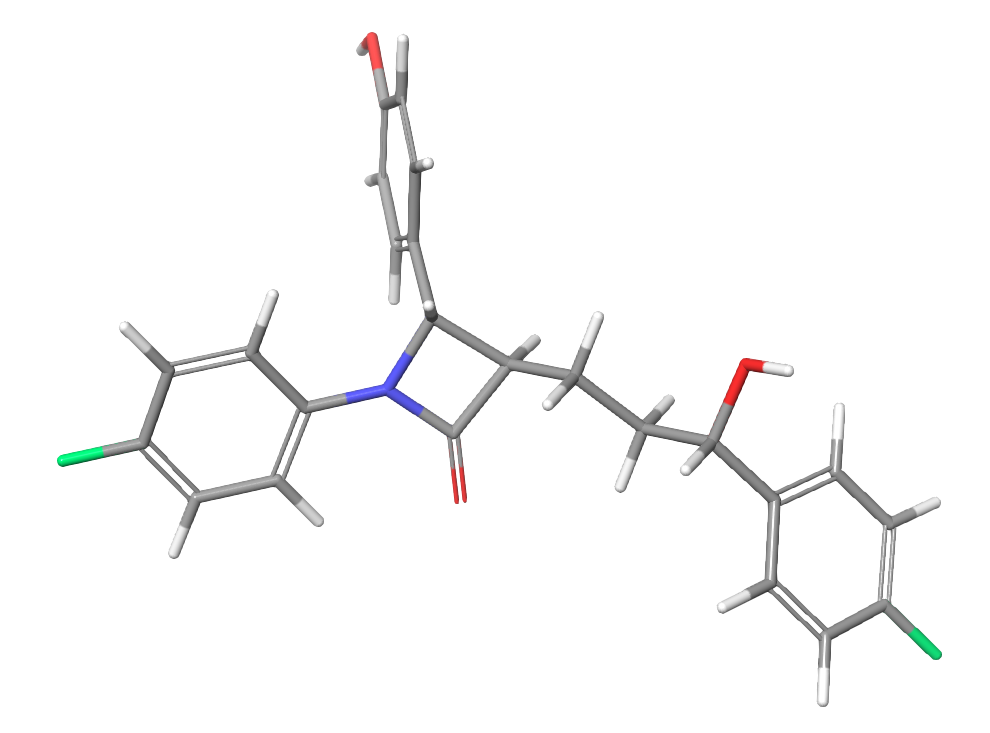


Conf2_10 (Boltzmann minimum)

C 5.43220 -1.26560 0.57140

C 4.93510 0.03520 0.57850

C 5.64880 1.02780 -0.08950

C 6.83030 0.73400 -0.75640

C 7.28660 -0.57050 -0.74680

C 6.60960 -1.58200 -0.09280

H 4.89580 -2.04820 1.09630

C 3.62720 0.34960 1.27310

H 5.28060 2.04580 -0.07870

H 7.39250 1.49960 -1.27510

F 8.43520 -0.86440 -1.39040

H 7.00330 -2.58990 -0.09920

H 3.46620 -0.40600 2.05310

O 3.62770 1.64710 1.85890

C 2.45180 0.29420 0.30270

C 1.10070 0.48090 0.98510

H 2.59780 1.06790 -0.45800

H 2.47850 -0.66680 -0.21460

C -0.06110 0.42900 0.00780

H 0.96700 -0.28760 1.75360

H 1.08200 1.44470 1.49800

H 4.32490 1.67700 2.52080

H 0.07840 1.14920 -0.80150

C -1.50700 0.50010 0.59590

C -0.47310 -0.93940 -0.52870

N -1.74680 -0.81220 -0.03530

O 0.07020 -1.83750 -1.13330

C -2.39110 1.63220 0.16340

H -1.50220 0.42280 1.68590

C -2.84660 -1.66860 0.01910

C -2.81190 1.77460 -1.15750

C -2.77840 2.60250 1.08530

C -3.55060 3.68940 0.70770

C -3.59040 2.85190 -1.54900

C -3.96090 3.81800 -0.61570

H -3.84670 4.43930 1.43010

O -4.72140 4.89540 -0.94020

H -2.47110 2.50860 2.12090

H -2.53660 1.03160 -1.89700

H -3.91340 2.94370 -2.58030

C -2.85240 -2.86370 -0.70640

C -3.94910 -1.33500 0.80630

C -3.94630 -3.71160 -0.63960

C -5.04620 -2.18100 0.87080

C -5.02630 -3.35650 0.14660

H -1.99790 -3.12350 -1.31390

H -3.95510 -0.41250 1.37070

H -5.90650 -1.93170 1.47760

F -6.09120 -4.18220 0.20800

H -3.96340 -4.64050 -1.19420

H -4.93910 4.86700 -1.87750

1. *ab initio* calculations at B3LYP / 6-31G*

**Input file**

<Settings>

title: Ezetimibe SRS

broadening: 5.0

minimum_wavenumber: 1100.0

maximum_wavenumber: 1800.0

temperature: 298.0

defined_scaling_factor: 0.970

boltzmann_cutoff: 10.0

print_csv spectra scaling_factor summary

<Experiments>

../../Experimental_DMSO/Ezetimibe_VCD_spectrum_averaged.csv

<Blank>

../../Experimental_DMSO/DMSO_blank_VCD_spectrum_averaged.csv

<Calculations>

../../Calculation_Gas/SRS_B3LYP

**Output file**

##########################################

## Cai•VCD analysis ##

## University of Cambridge, 2020 ##

##########################################

Input_Ezetimibe_SRS_B3LYP

minimum wavenumber: 1100.0

maximum wavenumber: 1800.0

Lorentzian broadening: 5.0

Temperature for Boltzmann averaging: 298.0 K

Defined scaling factor: 0.97

Print out graphs: True

Boltzmann Analysis: True

Boltzmann Analysis Energy cut-off: 10.0

Unique calculated structure criteria: Energy: 0.0001 Frequency: 2.0

Extreme scale factor warning range: 0.01

Insufficient information criterion for Cai.factor: 10

Printing spectra in .csv file

Printing scaling factor analysis in .csv file

Printing two-line summary in .csv file

Experimental data for single enantiomer:

(A) ../../Experimental_DMSO/Ezetimibe_VCD_spectrum_averaged.csv

Blank file:

../../Experimental_DMSO/DMSO_blank_VCD_spectrum_averaged.csv

Calculation files:

../../Calculation_Gas/SRS_B3LYP

Only one filename listed for calculations

which is a directory

Number of files: 130

Calculated data in Gaussian file

96 files rejected by energy cutoff

10 duplicate files removed

Unique Calculated Structures

Energy: -1406.946501 hartrees, 0.000 kJ/mol, Boltzmann Factor: 1.000 ../../Calculation_Gas/SRS_B3LYP/conf2_25_B3LYP_631GS

Energy: -1406.946372 hartrees, 0.339 kJ/mol, Boltzmann Factor: 0.872 ../../Calculation_Gas/SRS_B3LYP/conf2_104_B3LYP_631GS

Energy: -1406.945423 hartrees, 2.831 kJ/mol, Boltzmann Factor: 0.319 ../../Calculation_Gas/SRS_B3LYP/conf2_37_B3LYP_631GS

Energy: -1406.945216 hartrees, 3.374 kJ/mol, Boltzmann Factor: 0.256 ../../Calculation_Gas/SRS_B3LYP/conf2_1_B3LYP_631GS

Energy: -1406.945154 hartrees, 3.537 kJ/mol, Boltzmann Factor: 0.240 ../../Calculation_Gas/SRS_B3LYP/conf2_24_B3LYP_631GS

Energy: -1406.945023 hartrees, 3.881 kJ/mol, Boltzmann Factor: 0.209 ../../Calculation_Gas/SRS_B3LYP/conf2_18_B3LYP_631GS

Energy: -1406.944652 hartrees, 4.855 kJ/mol, Boltzmann Factor: 0.141 ../../Calculation_Gas/SRS_B3LYP/conf2_26_B3LYP_631GS

Energy: -1406.944540 hartrees, 5.149 kJ/mol, Boltzmann Factor: 0.125 ../../Calculation_Gas/SRS_B3LYP/conf2_41_B3LYP_631GS

Energy: -1406.944175 hartrees, 6.108 kJ/mol, Boltzmann Factor: 0.085 ../../Calculation_Gas/SRS_B3LYP/conf2_6_B3LYP_631GS

Energy: -1406.944096 hartrees, 6.315 kJ/mol, Boltzmann Factor: 0.078 ../../Calculation_Gas/SRS_B3LYP/conf2_73_B3LYP_631GS

Energy: -1406.944001 hartrees, 6.564 kJ/mol, Boltzmann Factor: 0.071 ../../Calculation_Gas/SRS_B3LYP/conf2_15_B3LYP_631GS

Energy: -1406.943734 hartrees, 7.266 kJ/mol, Boltzmann Factor: 0.053 ../../Calculation_Gas/SRS_B3LYP/conf2_78_B3LYP_631GS

Energy: -1406.943551 hartrees, 7.746 kJ/mol, Boltzmann Factor: 0.044 ../../Calculation_Gas/SRS_B3LYP/conf2_55_B3LYP_631GS

Energy: -1406.943341 hartrees, 8.298 kJ/mol, Boltzmann Factor: 0.035 ../../Calculation_Gas/SRS_B3LYP/conf2_77_B3LYP_631GS

Energy: -1406.943185 hartrees, 8.707 kJ/mol, Boltzmann Factor: 0.030 ../../Calculation_Gas/SRS_B3LYP/conf2_39_B3LYP_631GS

Energy: -1406.943071 hartrees, 9.006 kJ/mol, Boltzmann Factor: 0.026 ../../Calculation_Gas/SRS_B3LYP/conf2_45_B3LYP_631GS

Energy: -1406.942993 hartrees, 9.211 kJ/mol, Boltzmann Factor: 0.024 ../../Calculation_Gas/SRS_B3LYP/conf2_59_B3LYP_631GS

Energy: -1406.942983 hartrees, 9.238 kJ/mol, Boltzmann Factor: 0.024 ../../Calculation_Gas/SRS_B3LYP/conf2_92_B3LYP_631GS

Energy: -1406.942959 hartrees, 9.301 kJ/mol, Boltzmann Factor: 0.023 ../../Calculation_Gas/SRS_B3LYP/conf2_57_B3LYP_631GS

Energy: -1406.942951 hartrees, 9.322 kJ/mol, Boltzmann Factor: 0.023 ../../Calculation_Gas/SRS_B3LYP/conf2_16_B3LYP_631GS

Energy: -1406.942852 hartrees, 9.582 kJ/mol, Boltzmann Factor: 0.021 ../../Calculation_Gas/SRS_B3LYP/conf2_23_B3LYP_631GS

Energy: -1406.942746 hartrees, 9.860 kJ/mol, Boltzmann Factor: 0.019 ../../Calculation_Gas/SRS_B3LYP/conf2_31_B3LYP_631GS

Energy: -1406.942740 hartrees, 9.876 kJ/mol, Boltzmann Factor: 0.019 ../../Calculation_Gas/SRS_B3LYP/conf2_14_B3LYP_631GS

Energy: -1406.942724 hartrees, 9.918 kJ/mol, Boltzmann Factor: 0.018 ../../Calculation_Gas/SRS_B3LYP/conf2_33_B3LYP_631GS

Using all 24 unique conformations within energy cut-off

Defined scaling factor: 0.97

Single enantiomer result: Defined SF : 0.970 ; File (A) is assigned to the enantiomer calculated with Cai.factor 19

Single enantiomer blank: Defined SF : 0.970 ; File (A) is assigned to the enantiomer calculated with Cai.factor 26

Single enantiomer result: Opt. SF : 0.966 ; File (A) is assigned to the enantiomer calculated with Cai.factor 26

Single enantiomer blank: Opt. SF : 0.966 ; File (A) is assigned to the enantiomer calculated with Cai.factor 37

Experimental data for single enantiomer:

(A) ../../Experimental_DMSO/Ezetimibe_VCD_spectrum_averaged.csv

Blank file:

../../Experimental_DMSO/DMSO_blank_VCD_spectrum_averaged.csv

Single enantiomer summary

This is based on File (A) ( possible )

Using the blank data, File (A) is cautiously confident

Optimising the scale factor increases the confidence level: fairly confident

Overall Cai.factor is 32 which means fairly confident assignment


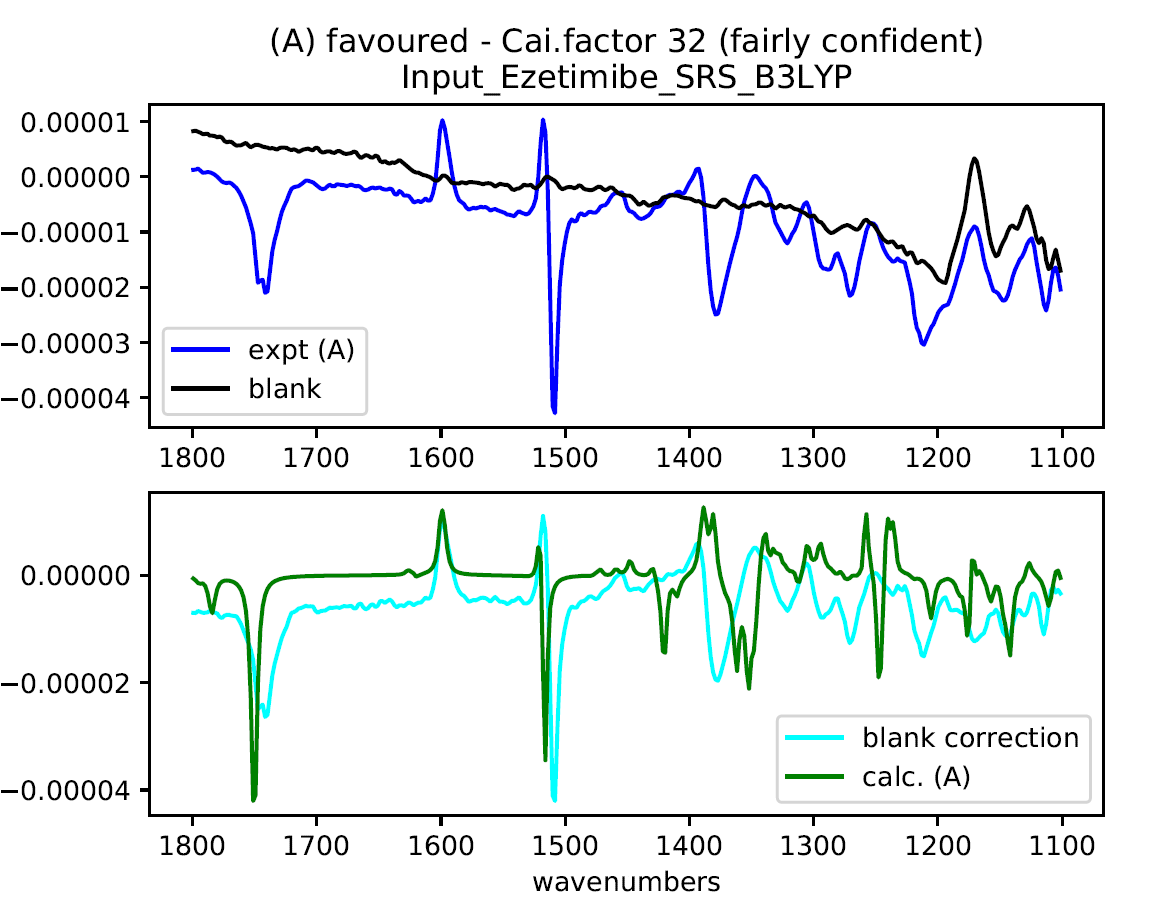


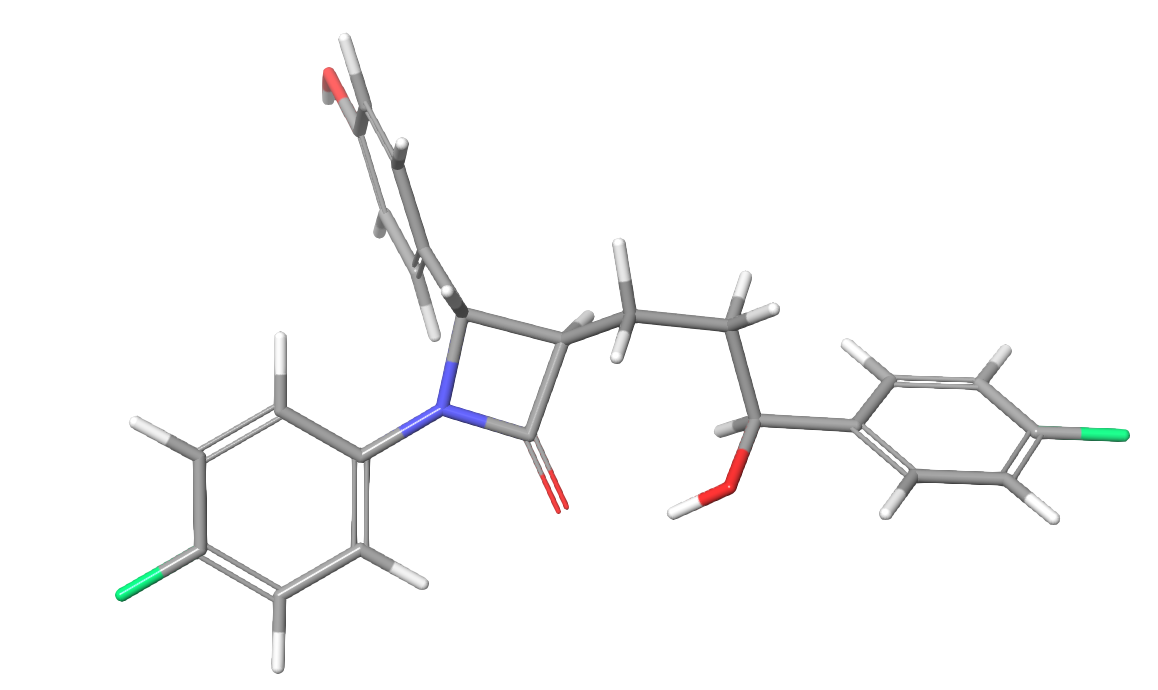


Conf2_25 (Boltzmann minimum)

C -5.46090 1.19900 0.44650

C -4.53170 0.28010 -0.05330

C -5.00060 -0.82080 -0.78230

C -6.36380 -1.01310 -1.00500

C -7.25830 -0.08360 -0.48710

C -6.82920 1.02300 0.23420

H -5.09820 2.06320 0.99150

C -3.04210 0.45530 0.20720

H -4.29360 -1.53860 -1.19340

H -6.73650 -1.85900 -1.57360

F -8.58260 -0.25980 -0.69940

H -7.55930 1.73070 0.61400

H -2.50900 0.29410 -0.74190

O -2.77450 1.76910 0.67720

C -2.53830 -0.58620 1.23340

C -1.07430 -0.40630 1.66830

H -3.17300 -0.52150 2.12550

H -2.68000 -1.59380 0.81800

C -0.05500 -0.41840 0.52190

H -0.97930 0.52860 2.23280

H -0.82000 -1.22110 2.35920

H -1.97640 2.06960 0.20240

H -0.32410 -1.16440 -0.23590

C 1.47170 -0.52620 0.88930

C 0.32780 0.92470 -0.11930

N 1.66340 0.74530 0.14650

O -0.27210 1.85390 -0.64110

C 2.24000 -1.72960 0.40680

H 1.63690 -0.37780 1.96390

C 2.79120 1.57430 -0.00430

C 2.45930 -1.96400 -0.95660

C 2.71740 -2.67320 1.32710

C 3.38320 -3.82150 0.90920

C 3.13000 -3.10510 -1.38920

C 3.59240 -4.04120 -0.45620

H 3.75210 -4.55130 1.62270

O 4.25630 -5.17820 -0.81610

H 2.56440 -2.50860 2.39160

H 2.11470 -1.24120 -1.69150

H 3.29860 -3.26720 -2.45230

C 4.02450 1.17890 0.53220

C 2.68300 2.79740 -0.68760

C 5.14380 1.99750 0.39050

C 3.80240 3.61360 -0.82630

C 5.01680 3.20350 -0.28670

H 4.11510 0.23060 1.05050

H 1.72550 3.09950 -1.09370

H 3.74090 4.56350 -1.34700

F 6.10010 3.99740 -0.42500

H 6.10680 1.70810 0.79790

H 4.33880 -5.20750 -1.78210
